# A novel vector for transgenesis in the rat CNS

**DOI:** 10.1186/s40478-017-0484-y

**Published:** 2017-11-21

**Authors:** T. Peter Lopez, Kurt Giles, Brittany N. Dugger, Abby Oehler, Carlo Condello, Zuzana Krejciova, Julian A. Castaneda, George A. Carlson, Stanley B. Prusiner

**Affiliations:** 1 Institute for Neurodegenerative Diseases, Weill Institute for Neurosciences, University of California, San Francisco, Sandler Neurosciences Center, 675 Nelson Rising Lane, San Francisco, CA 94158 USA; 2 Department of Neurology, University of California San Francisco, San Francisco, CA 94158 USA; 3 Department of Biochemistry and Biophysics, University of California, San Francisco, CA 94158 USA

**Keywords:** Scrapie, Vector, *Prnp*, Neurodegenerative disease

## Abstract

The larger brain of the rat enables a much greater repertoire of complex behaviors than mice, likely making rats preferential for investigating neurodegeneration. Because molecular tools for specific expression of transgenes in the rat brain are sparse, we chose *Prnp* encoding the prion protein (PrP) to develop a novel vector to drive transgene expression in the rat brain. We compared the rat *Prnp* sequence with mouse and Syrian hamster *Prnp* sequences, identifying conserved genetic elements and hypothesizing that these elements would be able to drive neuronal transgene expression. We investigated this by generating a vector termed RaPrnp that encompasses portions of the rat *Prnp* gene. Importantly, we replaced the rat *Prnp* open reading frame (ORF) with a cloning site for rapid and seamless In-Fusion cloning. To validate the in vivo neuronal specificity of the RaPrnp vector in rats, we generated stable RaPrnp-LacZ/enhanced green fluorescent protein (EGFP) transgenic (Tg) rat lines, which led to robust LacZ activity and high EGFP fluorescence in the central nervous system of embryos and adult animals. Next, we restored the rat *Prnp* ORF and generated multiple Tg(RaPrnp-PrP) lines, demonstrating that overexpression of *Prnp* accelerates the onset of scrapie. While the incubation time in wild-type (WT) rats was 175 ± 3 days post inoculation (dpi), one line, Tg2919, overexpressed RaPrP^C^ at 4.4-fold and exhibited a reduced incubation time of 149 ± 2 dpi. The second line, Tg2922, overexpressed RaPrP^C^ at 9.7-fold compared with WT animals and had an incubation time of 112 ± 0 dpi. Tg2922 rats inoculated with rat RML showed extensive vacuolation of the brainstem in contrast to WT and Tg2919 animals in which vacuolation was most prominent in the hippocampus and striatum as well as the motor and sensory cortices. It is possible that construction of Tg rats with modified phenotypes will prove more advantageous than mice for neurodegeneration studies.

**Electronic supplementary material:**

The online version of this article (10.1186/s40478-017-0484-y) contains supplementary material, which is available to authorized users.

## Introduction

Neurodegenerative disorders including Alzheimer’s disease (AD), the tauopathies, Parkinson’s disease (PD), multiple system atrophy (MSA), and PrP prion diseases such as Creutzfeldt–Jakob disease (CJD) are all progressive illnesses that cause increasing central nervous system (CNS) dysfunction and are eventually fatal (see [14]). In each of these maladies, there is increasing evidence that one or two proteins, termed Aβ, tau, α-synuclein, and PrP^C^, undergo a conformational change enriched for β-sheet that leads to the self-propagation and accumulation of prions within the CNS. The accumulation of a particular prion correlates with the onset of neurological dysfunction, which often manifests as motor deficits and/or dementia. For decades, mice have been the preferred organism to model neurodegenerative disease (ND) and have provided key mechanistic insights into these delayed onset illnesses. Given the complexity of the human NDs, it seems that better animal models are likely to represent seminal advances in the discovery of effective therapeutics for these disorders.

Although transgenic (Tg) mice have been invaluable tools in dissecting the biology and pathogenesis of many of the NDs [13], there are numerous vexing questions that may require more faithful animal models. With advances in transgenesis, the laboratory rat is slowly gaining a more important role in modeling NDs. Notably, rats have been used extensively for modeling neuropsychiatric and behavioral disorders because their CNS is sufficiently complex to reflect such illnesses. Moreover, the rat brain has nearly 200 million neurons, which is three-fold greater than the brains of mice [7]. The larger brains of rats also enable better microdissection for biochemical and molecular characterization. While MRI and PET can be performed on mice, the larger brain volume of the rat provides greater resolution for extensive CNS imaging. In addition, rats produce ten times more cerebrospinal fluid (CSF) than mice, making serial sampling of CSF possible [6, 11]. For these and other reasons, the rat may prove to be superior for modeling human NDs despite the increased cost of animal husbandry required because of its larger size.

Although Tg rats are potentially more advantageous than mice to model ND, rat-specific tools for transgene delivery to the CNS are scarce. *Huntingtin* and *Synapsin I* rat promoters have previously been used, but their limited spatial and temporal range of expression in the CNS has proved inadequate for generating AD models [8, 21]. On the other hand, mouse *Thy-1* and *Prnp* promoters have led to modest AD phenotypes in rats [3, 10]. Pronuclear microinjection of a large bacterial artificial chromosome (BAC) containing wild-type (WT) human α-synuclein was used to generate rats with PD-like symptoms [12]. However, overexpression was limited, as human α-synuclein expression levels were maximally ~2–3 fold higher than endogenous rat α-synuclein [12]. Moreover, routine generation of Tg rats by BAC transgenesis has proved not to be ideal because such large constructs are often inefficient, and foreign DNA may not contain the optimal regulatory elements for sustained expression in the rat brain. Given that some NDs may require higher gene expression to advance disease progression during the life span of the rat, the development of a pan-neuronal Tg vector that delivers high expression in multiple brain regions might prove more useful.

Previously, we developed the cos.Tet Tg vector, which contains ~43 kb of Syrian hamster (SHa) regulatory elements to drive transgene expression in mice [17]. While cos.Tet-based transgenes led to appreciable levels of overexpression, the size of the vector made it challenging for cloning in large DNA fragments or efficient transgenesis. Subsequently, the “half-genomic PrP” [5] and MoPrP.Xho vectors were developed [1]. Together, these vectors have been used to generate dozens of Tg mouse lines [22]. On this background, we set out to create a novel vector that was (1) amendable for seamless cloning of genetic cargo, (2) efficient for generating Tg rats, and (3) able to deliver high levels of expression in neurons throughout the rat brain to study ND. Such a tool would be useful to the scientific community in developing a range of Tg rat models.

In this study, we identified conserved promoter elements in rodent *Prnp* genes and incorporated them into a vector, termed RaPrnp. We validated RaPrnp-mediated expression in rodent cells and studied spatiotemporal expression in the CNS of Tg rats. To test whether this vector could be used to modulate ND in rats, we generated animals  overexpressing the normal rat prion protein isoform (PrP^C^), the overexpression of which accelerates prion disease by increasing the propagation of the infectious isoform, PrP^Sc^. The RaPrnp vector need not be restricted to PrP prion diseases; certainly, it should find utility in studies of neurodegeneration caused by Aβ, tau, or α-synuclein prions.

## Materials and methods

### Construction of the RaPrnp vector

VISTA software (http://genome.lbl.gov/vista/index.shtml) was used to align the rat *Prnp* locus with defined intronic and exonic regions (~23 kb; University of California, Santa Cruz, Genome Browser assembly ID: rn6; Entrez Gene: 24686) with mouse and SHa *Prnp*. Conserved regions were defined using a scanning 100 bp window with an identity greater than 50%. A rat bacterial artificial chromosome (BAC) template (Children’s Hospital Oakland Research Institute, CH230-380 M13) containing the rat *Prnp* locus was used for polymerase chain reactions (PCR) to amplify two fragments. The first fragment is Region I, which contains 6 kb of upstream sequence, to include elements of the rat *Prnp* promoter, Exon 1, Intron 1, and Exon 2. The second fragment, based on Region III, was PCR amplified; the open reading frame (ORF) of E3 was removed and a unique XhoI site was introduced in its place followed by the 3’UTR and 2.3 kb of downstream sequence. Importantly, we incorporated 15 bp In-Fusion flanking homology arms in Regions I and III and a linearized pUC19 backbone. With the addition of In-Fusion enzyme (Clonetech), all three fragments were combined to generate the RaPrnp vector. PE300 sequencing of the cos.Tet vector was done on a MiSeq instrument (Illumina) by the Mouse Biology Program at the University of California, Davis.

### Cloning of transgene constructs

The RaPrnp vector was digested with XhoI and gel purified. Next, *LacZ-T2A* or *T2A-EGFP* fragments were PCR amplified from *pGfaABC1D-nLac* [9] and *p799-IRES-EGFP* (a gift from Jonathan Rubenstein) vectors, respectively. A dual *LacZ-T2A-EGFP* reporter cassette was created by overlap PCR. The rat *Prnp* ORF was PCR amplified from the original rat BAC template. Importantly, we generated flanking homology arms for the *LacZ-T2A-EGFP* and rat *Prnp* ORF to the XhoI linearized RaPrnp vector. Finally, In-Fusion cloning was used to introduce the *LacZ-T2A-EGFP* (*LacZ/EGFP*) cassette or rat *Prnp* ORF into the RaPrnp vector. Primer sequences used to amplify products are listed in Online Resource, Additional file 1: Table S1.

### Expression of the RaPrnp-LacZ/EGFP vector in cells

One μg of RaPrnp-LacZ/EGFP plasmid was transfected via X-tremeGENE HP DNA transfection reagent (Roche) into ~3 × 10^5^ cells per well in a six-well dish. Two days post-transfection, live EGFP fluorescence was captured by an EVOS Cell Imaging System using a 20× objective.

### Generation of Tg rats

Methods supporting the preparation and generation of Tg rats were modified from Filipiak et al. [4]. Sprague Dawley (SD) rats were purchased from Charles River and allowed to acclimatize for 3–5 days in our facility prior to hormonal treatment. Rats were kept on a 12-h light/dark cycle (lights on 2 a.m. to 2 p.m.). Recipient SD female rats (9–10 weeks old) were synchronized for estrus  by injection with 0.2 ml (40 μg) Luteinizing Hormone-Releasing Hormone Analog (SIGMA, L4513) at 8 a.m. 4 days before mating with vasectomized SD males (2–8 months old) to induce pseudopregnancy. Pseudopregnancy was confirmed the following day by detecting the remains of a mating plug in recipients by an otoscope. Donor SD females (26–28 days old) were superovulated by injecting 0.2 ml (20 IU) of pregnant mare serum gonadotropin at 8 a.m. 2 days before mating followed by 0.2 ml (30 IU) human chorionic gonadotropin at 10 a.m. immediately before mating with donor males (2–8 months old). The day after mating, donor females were examined for the presence of mating plugs and euthanized by CO_2_ followed by cervical dislocation. Oviducts were collected and placed in room temperature M2 media (Sigma Aldrich, M7167); the cumulus mass was released by tearing the ampulla. To detach the cumulus cells from the embryos, the cumulus mass was transferred to a drop of M2 containing hyaluronidase (1 mg/ml, Sigma, H4272) for 5–7 min. Embryos were serially washed in 5–7 drops of M2 media and then transferred into pre-equilibrated M16 media (Sigma Aldrich, M7292) and incubated at 37 °C; 5% CO_2_ for 60 min. RaPrnp-LacZ/EGFP or RaPrnp-PrP transgenes were excised with NotI and gel purified to remove pUC19 backbone sequences. Gel-extracted fragments were purified with a Zymoclean large fragment DNA recovery kit (Zymo Research, D4046) and eluted into EmbryoMax injection buffer (Milipore, MR-095-10F). DNA was dialyzed on a DNA dialysis filter membrane (Millipore, VSWP02500) for 60–90 min in water for embryo transfer (Sigma, #W1503). A Nanodrop spectrophotometer (Thermo Scientific) was used to assess the concentration and purity (260/280 ratio of ≥1.8 and 260/230 ratio of 1.8–2.2) of dialyzed DNA sample. A sample of the resulting DNA was run on a 0.8% agarose gel to confirm a single band was visible. Transgenes were microinjected at 1–2 ng/μl into the pronuclei of one-cell stage rat zygotes. Because the cellular and pronuclear membranes of rat zygotes are highly elastic, microinjection needles were pulled longer and thinner (0.4–0.6 μm tip with 10 mm taper) than needles used for microinjection into mouse zygotes. Following microinjection, viable rat embryos were incubated in pre-equilibrated M16 media at 37 °C; 5% CO_2_ for 30–60 min and transferred into the oviducts of pseudopregnant recipient females. All recipient females were administered analgesics, including meloxicam 2 mg/kg subcutaneously, sustained-release (SR) buprenorphine 1 mg/kg, and a local block of bupivacaine ≤ 7 mg/kg perioperative. The meloxicam and SR buprenorphine were continued pro re nata. Pups were screened for the presence of RaPrnp-LacZ/EGFP or RaPrnp-PrP transgenes by PCR with a RaPrnp forward genotyping primer and either a LacZ-T2A-EGFP or rat *Prnp* ORF reverse genotyping primer (Online Resource, Additional file 1: Table S1). Potential founder animals were mated to WT SD rats to establish the lines.

### *Prnp* copy number detection by droplet digital PCR

Rat genomic DNA (gDNA) was purified from ear or tail samples from Tg(RaPrnp-PrP) rats and diluted to 100 ng/μl. One μg of gDNA was digested with MseI (New England Biolabs R0525L) for 1 h at 37 °C. PCR reactions were set up with digested gDNA, ddPCR SuperMix: non-UTP (BioRad #1863024), *Prnp*-FAM (BioRad #10042958) target, and *Rpp30*-Hex (BioRad #10042961) reference copy number assay kits. PCR reactions and droplet reader oil (BioRad #1863004) were combined and then added to a QX200 droplet generator instrument (BioRad). Droplets underwent thermocycling in a C1000 instrument (BioRad) and read via a QX200 droplet reader. Copy number variation (CNV) analysis was done using QuantaSoft software (BioRad) by comparing the concentration of *Prnp* target (A) to the concentration of the *Rpp30* (B) reference loci in gDNA samples. N_B_ = refers to 2 copies of *Rpp30* in the rat genome. CNV = A/B*N_B_. Founder transgene copy number was determined by the subtraction of WT *Prnp* copies (2) from the total *Prnp* copy number derived from the ddPCR assay.

### Clinical assessment of animals

All rats were examined at least once per day by a trained animal health technician for general appearance, activity level, hydration, body condition, abnormal posture, porphyrin staining, nature of ambulation, respiration quality, and qualitative food and water intake. A licensed veterinary technician or a veterinarian examined animals displaying any abnormal clinical sign. These included ataxia, bradykinesia, lethargy, hair loss, moribund state, porphyrin staining, clasping, and poor hair coat. Clinically sick animals were gently prodded to assess quality of ambulation and were briefly suspended by the tail to perform the hind-leg clasping reflex. Animals displaying two or more clinical signs were identified, and if their clinical condition did not change or deteriorated within 24 h, then the animal(s) was euthanized to define the incubation period in these studies.

### Neuropathology of prion-infected rats

Formalin-fixed hemi-brains were coronally coarse-cut and embedded into a paraffin block. After paraffin processing and embedding, sections were cut using a microtome set at an 8 μm thickness. Brain sections were then mounted on positively charged glass slides. These were then deparaffinized and stained with hematoxylin and eosin (H&E) or processed for immunohistochemistry. For immunofluorescent procedures, after removal of paraffin with xylenes and a graded series of alcohols, tissue sections were subjected to antigen retrieval with hydrolytic autoclaving (121 °C for 10 min in citrate buffer). After antigen retrieval, sections were incubated in 10% normal goat serum (Vector Laboratories, Inc., Burlingame, CA) made in 0.1 M PBS containing 0.2% Tween (PBST) for 1 h. After washing in PBST, slides were incubated in a primary antibody cocktail containing a 1:250 dilution of a rabbit PrP antibody (Abcam, ab52604) and 1:500 dilution of chicken GFAP (Abcam, ab4674) overnight at room temperature. The following day, sections were washed in PBST and incubated in a cocktail of specific secondary antibodies including goat anti-rabbit Alexa Fluor 488 (Thermo Fisher, Waltham, MA) and anti-chicken Alexa Fluor 647 (Thermo Fisher, Waltham, MA) diluted at 1:500 for 2 h at room temperature. Sections were then rinsed in PBST followed by addition to ddH_2_0 and subjected to a Hoechst stain (Invitrogen, Carlsbad, CA) for 10 min.

### Data availability

All data generated or analyzed during this study are included in this article and its Online Resource.

## Results

### Comparison of three rodent *Prnp* homologs for the generation of a Tg vector

**Fig. 1:**
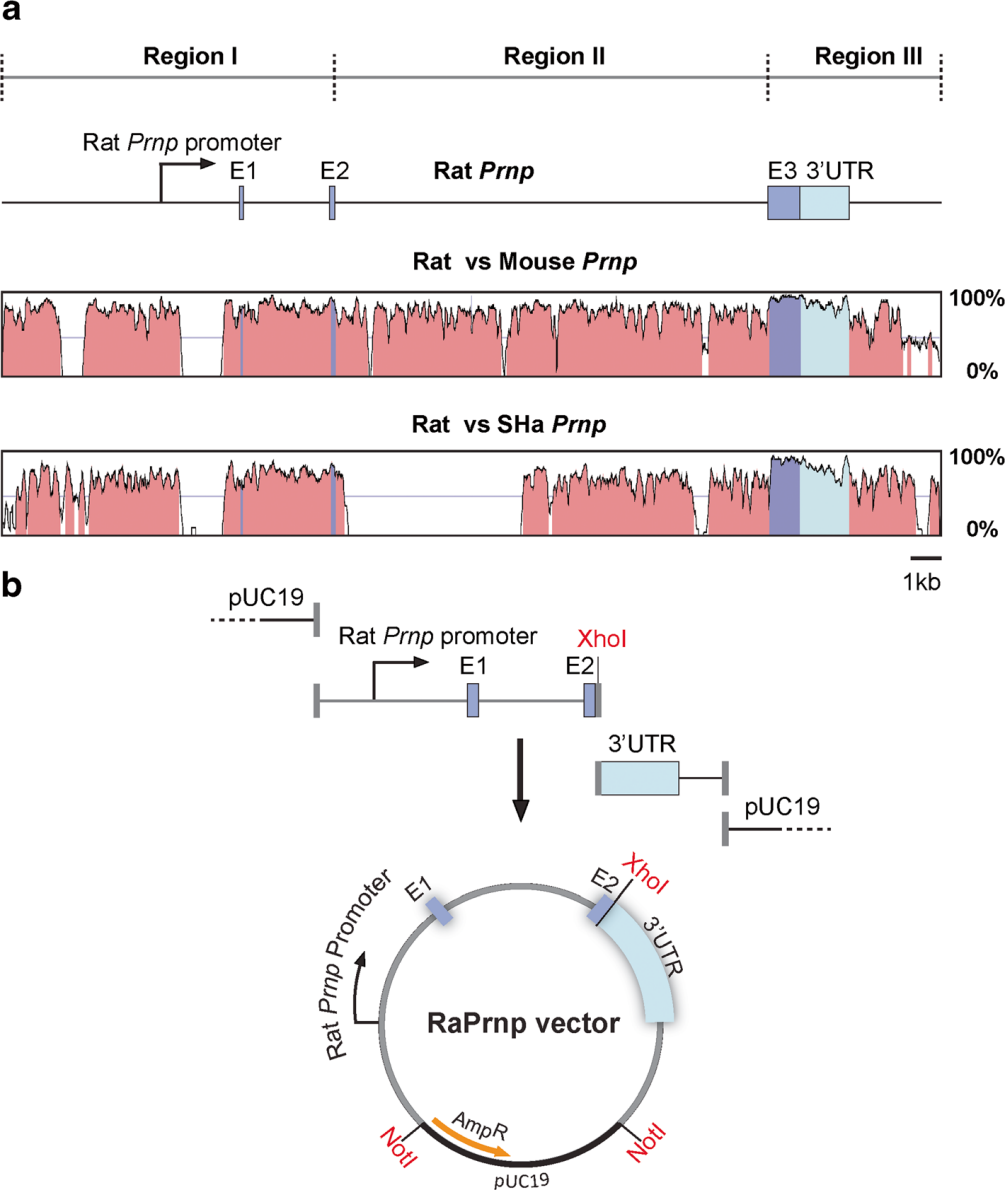
Generation of a Tg vector based on conserved *Prnp* genomic elements**.** (**a**) Schematic of the rat *Prnp* genomic locus compared with mouse and Syrian hamster (SHa) *Prnp* using the Vista alignment tool. We define the rat *Prnp* locus in three regions: Region I spans a 6 kb upstream sequence, Exon 1 (E1), Intron 1, and Exon 2 (E2). Region II represents Intron 2. Region III defines Exon 3 (E3), the 3’UTR, and 2.3 kb of downstream sequence. Using a sliding window of contiguous regions of 100 bp with an identity greater than 50%, graphical outputs were generated comparing genomic position (x-axis) to percent identity (y-axis). *Prnp* genetic elements with high similarity are color coded: dark blue = exons, light blue = UTR, and red = non-coding sequence/intergenic region. (**b**) Schematic of the cloning steps to generate the RaPrnp vector. The RaPrnp vector includes Region I and Region III with E3 removed and replaced with an XhoI site but maintains the 3’UTR and 2.3 kb downstream sequence. Gray vertical bars refer to the 15 bp In-Fusion homology arms. NotI sites were incorporated at the ends of the RaPrnp transgene to remove the pUC19 backbone. The AmpR resistance cassette is designated by the orange curved arrow

Based on the success of the cos.Tet and MoPrP.Xho vectors for the generation of Tg mice [1, 15, 20, 22], we compared the *Prnp* genomic sequence of the rat with that of the mouse and Syrian hamster to identify conserved DNA sequences. We analyzed sequences with a scanning window of 100 bp for an identity greater than 50%. We identified the following regions: a sequence spanning a 6-kb upstream fragment, Exon 1, Intron 1, and Exon 2 (Region I); Intron 2 (Region II); and Exon 3/3’UTR and a 2.3 downstream sequence containing putative polyadenylation signals (Region III) (Fig. 1a). Because rats and mice only diverged ~10 million years ago [18], their sequences show a high degree of similarity. The largest difference occurs in Region I, which contains two indels of 0.6 and 1.1 kb (Fig. 1a). Rats diverged from hamsters ~25 million years ago [18], and in addition to a 1.1 kb indel in Region I, there is a 4.5 kb indel in Region II. Thus, we created a ~13.3 kb vector combining Regions I and III, the most highly conserved genetic elements of the rat *Prnp* locus.

### Generation of a rat vector for transgene expression in the CNS

We PCR amplified rat *Prnp* Region I and III fragments to contain overlapping 15 bp homology arms for In-Fusion cloning to each other and a pUC19 backbone. The endogenous *Prnp* ORF was removed from Region III and replaced by an XhoI site for cloning of genetic cargo, while NotI sites were added to remove the transgene from the pUC19 backbone (Fig. 1b). Finally, an ~13.3 kb construct was assembled by an In-Fusion reaction containing pUC19, Region I, and Region III fragments. Hereafter, we refer to this construct as the RaPrnp vector (Fig. 1b). The complete sequence of this vector is located in the Online Resource, Additional file 1: Supplementary Information 1.

### In vivo and in vitro validation of the RaPrnp vector

**Fig. 2:**
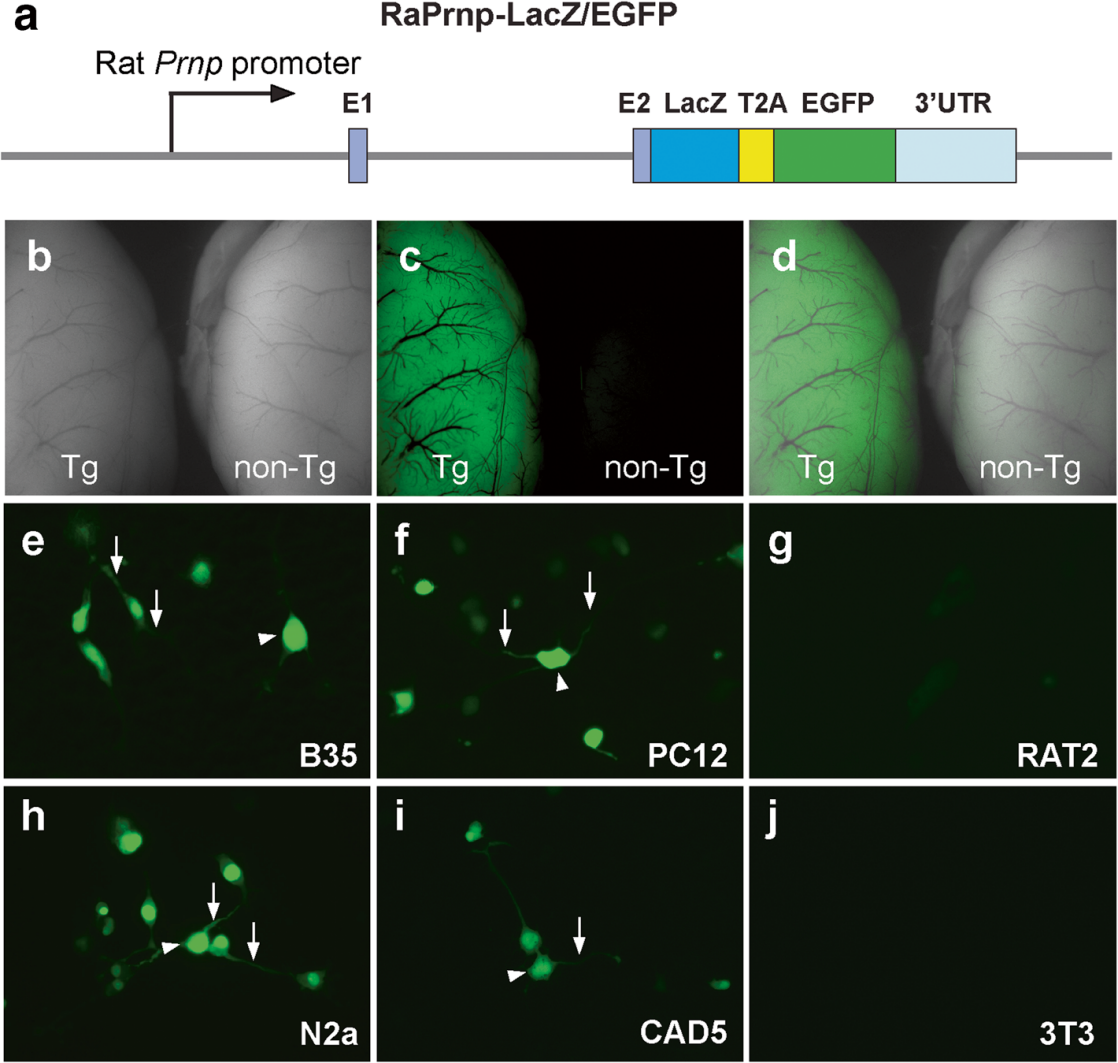
In vivo and in vitro validation of the RaPrnp vector. (**a**) A LacZ/EGFP dual-reporter cassette was cloned into the RaPrnp vector via In-Fusion cloning. Schematic of the RaPrnp-LacZ/EGFP construct following NotI digestion. (**b**) Brightfield, (**c**) EGFP fluorescence signal, and (**d**) merge of brains from 2-month-old Tg12084 rats. Left: Tg; right: non-Tg. Rat (**e**–**g**) and mouse (**h**–**j**) cells were transfected with the RaPrnp-LacZ/EGFP construct. Neuronal rat (e and f) and mouse (h and i) cells were transfected along with rat (**g**) and mouse (**j**) fibroblasts. EGFP fluorescence was detected in B35 (**e**), PC12 (**f**), N2a (**h**), and CAD5 (**i**) neuronal cell lines. A few transfected RAT2 fibroblast cells had low EGFP fluorescence (**g**). Mouse 3T3 cells transfected with the RaPrnp-LacZ/EGFP vector did not support EGFP fluorescence (**j**). Arrowheads = cytoplasm; arrows = neurite-like extensions

To determine if RaPrnp drives gene expression in vivo, we cloned in a dual reporter cassette containing the LacZ gene followed by a T2A ribosomal skipping sequence and an EGFP ORF (Fig. 2a) into the XhoI site of RaPrnp to generate a RaPrnp-LacZ/EGFP construct. We microinjected this construct into SD rat zygotes. Approximately 65% of microinjected eggs survived, and ~10% of the eggs implanted into surrogate dams yielded live births (Table 1). From 13 live births, we obtained two founders that stably transmitted the RaPrnp-LacZ/EGFP transgene to their progeny (Table 1). These two independent founder lines, Tg12084 and Tg12085, were backcrossed to WT SD rats. Tg12084 rat brains displayed high EGFP fluorescence in young adult animals at 2 months of age compared with non-Tg animals, which lacked EGFP fluorescence (Fig. 2b–d). In addition to these findings, we transfected the RaPrnp-LacZ/EGFP construct into various rodent cell lines: rat neuroblastoma (B35), pheochromocytoma (PC12), rat embryonic fibroblast (RAT2), mouse neuroblastoma (N2a), catecholaminergic (CAD5), and mouse embryonic fibroblast (3T3) (Fig. 2e–j). Two days post-transfection, we observed strong EGFP fluorescence in the cytoplasm and moderate fluorescence in the neurite-like extensions in B35, PC12, N2a, and CAD5 cells (Fig. 2e, f, h, and i). Conversely, in the non-neuronal cell lines, we found a few modestly EGFP-positive RAT2 cells, while the transfected 3T3 cell line did not reveal any EGFP fluorescence (Fig. 2g and j). Because RaPrnp was capable of facilitating gene expression in mouse neuronal cell lines (Fig. 2h and i), we determined that murine regulatory elements of *Prnp* were preserved in this vector.

**Table 1:** Transgenesis in Sprague Dawley (SD) rats

Transgene	Number of Eggs			Births	Potential founders	# of Potential founders bred	# of Founders
	Injected	Survived	Implanted				
RaPrnp-LacZ/EGFP	192	124	116	13	2	2	2
RaPrnp-PrP	667	543	489	64	15	7	4

### RaPrnp-mediated CNS expression during rat development

**Fig. 3:**
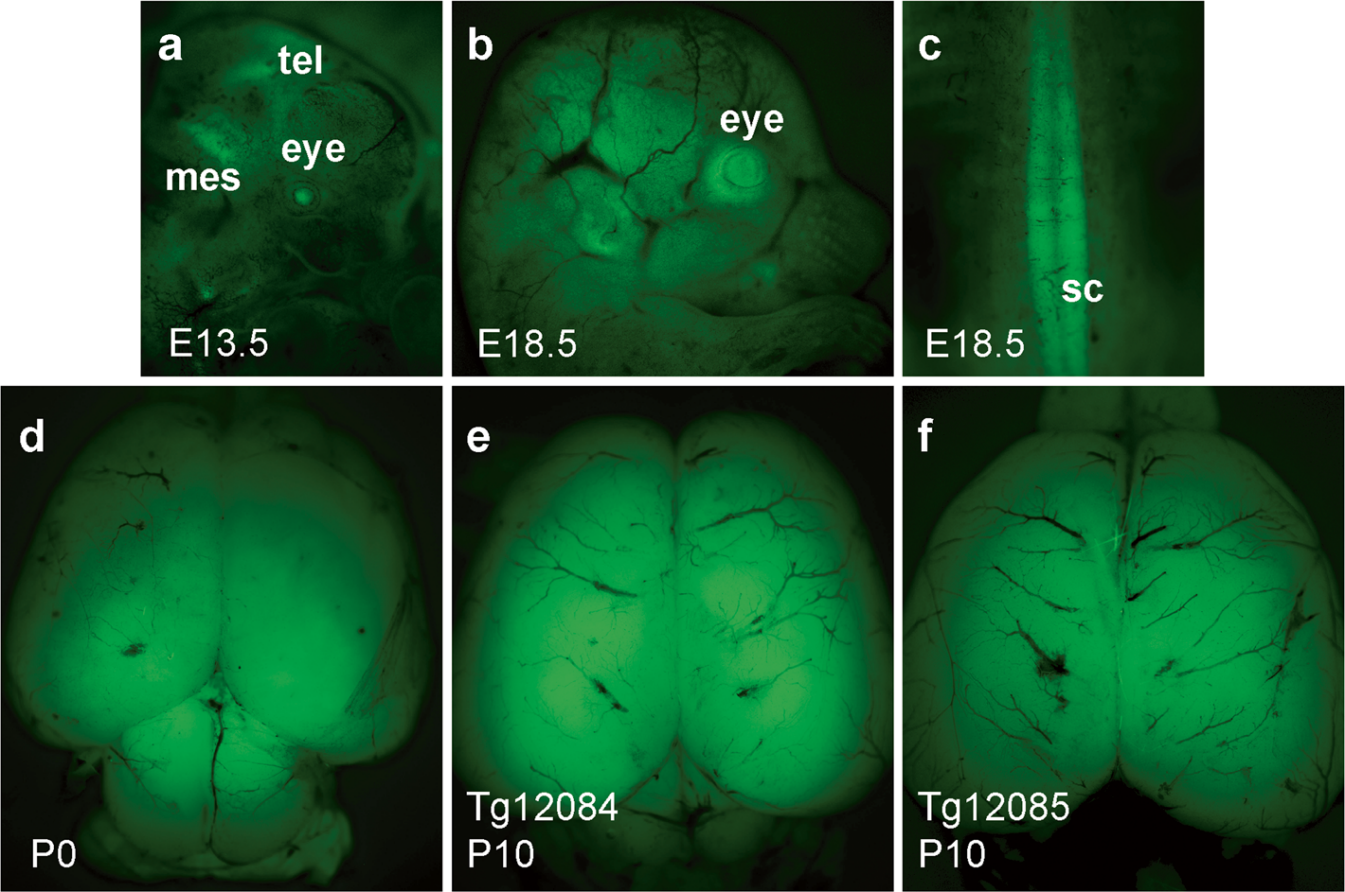
Developmental transgene expression in Tg(RaPrnp-LacZ/EGFP) embryos and neonates**.** EGFP fluorescence in Tg12084 (**a**–**e**) and Tg12085 (**f**) rats. (**a**) EGFP fluorescence is detected in the mesencephalon (mes), telencephalon (tel), and eye in E13.5 rat embryos. (**b**) EGFP is widely expressed in the brain, eye, and (**c**) spinal cord (sc) of E18.5 embryos. EGFP expression is observed throughout the brains of P0 (**d**) and P10 neonates (**e** and **f**)

Having observed expression in 2-month-old RaPrnp-LacZ/EGFP Tg rats, we sought to determine if the RaPrnp vector supported gene expression during embryogenesis and at early postnatal stages. For the purpose of staging during development and birth, we refer to the day a mating plug is observed as embryonic day 0.5 (E0.5), while the day of birth for neonatal pups is indicated as postnatal day 0 (P0), respectively. At the E9.5 time point, EGFP fluorescence was not apparent in either line; however, at the E13.5 time point, the Tg12084 line showed in vivo EGFP fluorescence in the mesencephalon, telencephalon, and eye (Fig. 3a). Additionally, in these animals, EGFP fluorescence continued to expand globally in the brain and spinal cord at E18.5, P0, and P10 (Fig. 3b–e). In contrast to the Tg12084 line, we did not observe EGFP fluorescence in the CNS of Tg12085 rats until P10 (Fig. 3f).

### RaPrnp drives transgene expression in adult rats

**Fig. 4:**
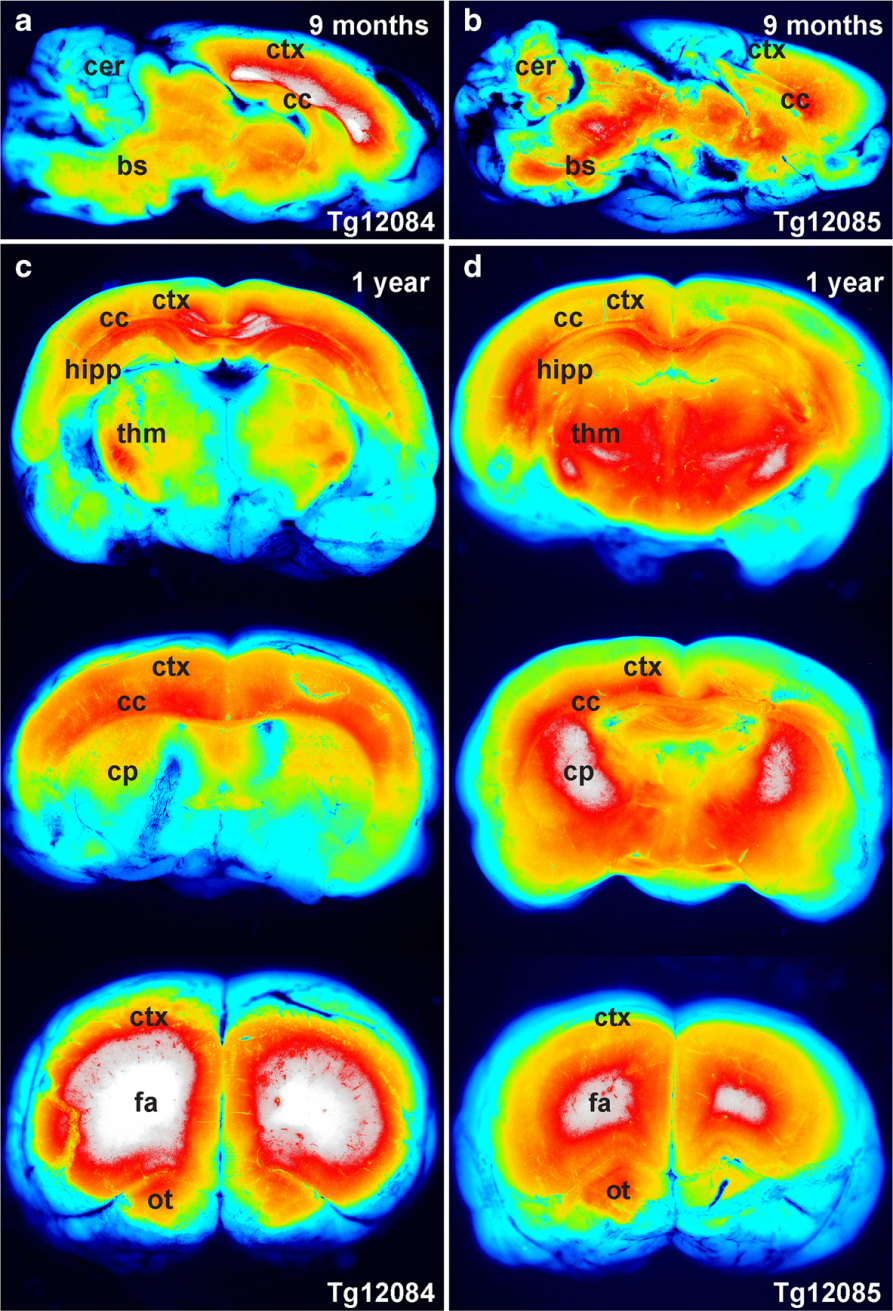
Spatial expression of RaPrnp-driven transgenes in adult rat brains. Fluorescence intensity in 9-month (**a** and **b**) and 1-year-old (**c** and **d**) rat brains. (**a**) Sagittal hemisphere of a Tg12084 rat brain demonstrates global EGFP fluorescence with peak fluorescence in the forebrain. (**b**) Sagittal hemisphere of a Tg12085 rat brain shows similar global EGFP signal, but the fluorescence is stronger in the brainstem, posterior, and midbrain compared with Tg12084 rats. Coronal serial slices through the brains of Tg12084 (**c**) and Tg12085 (**d**) rats show widespread EGFP signal. Top slices = midbrain. Middle slices = midbrain to forebrain. Bottom slices = forebrain. Brain stem (bs), caudoputamen (cp), cortex (ctx), corpus callosum (cc), cerebellum (cer), anterior forceps (fa), hippocampus (hipp), olfactory tuberde (ot), and thalamus (thm). Heat intensity maps of EGFP fluorescence depict low to high expression with a color gradient of blue, turquoise, green, orange, red, and white

**Fig. 5:**
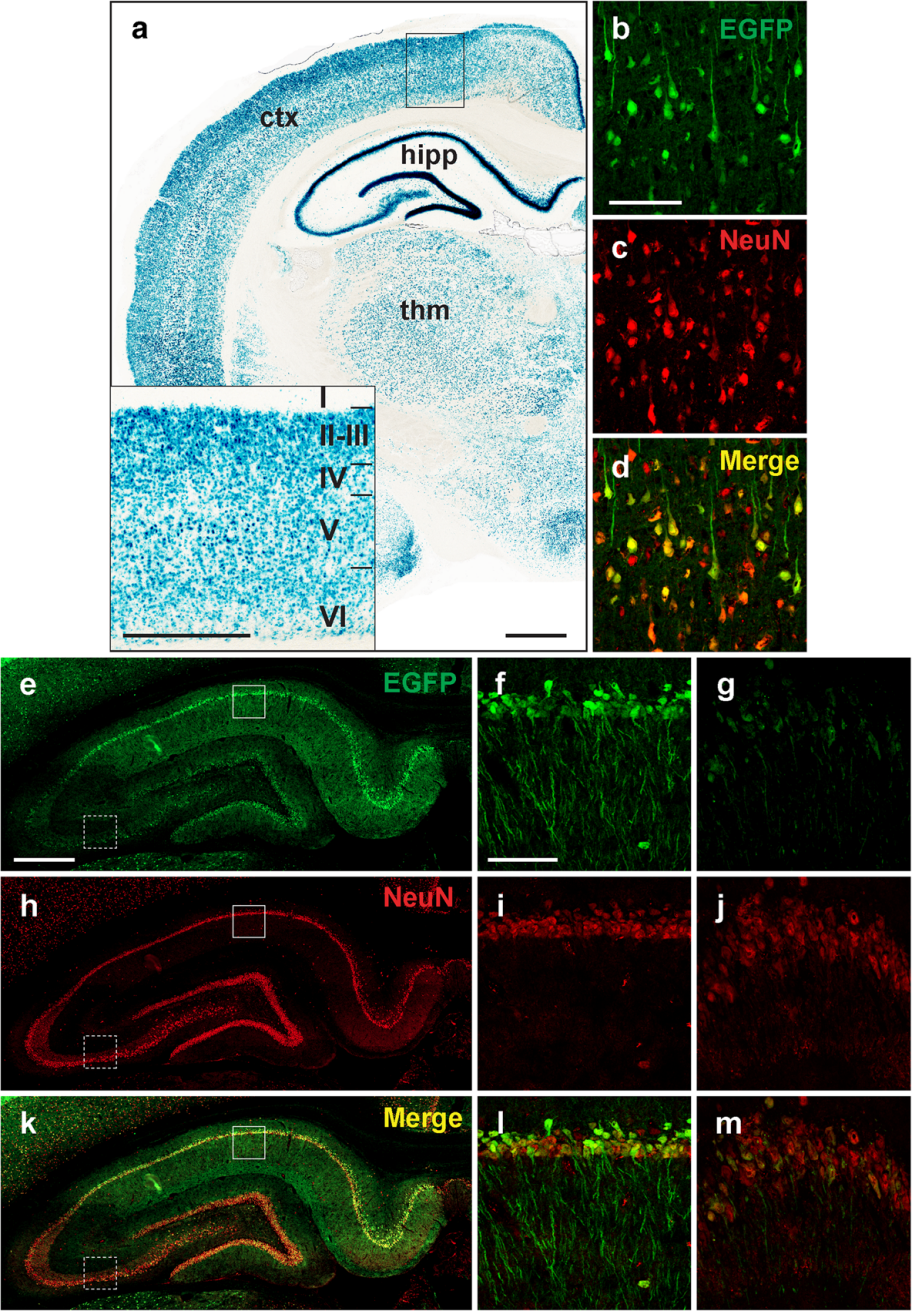
RaPrnp vector drives expression in rat neurons. Visualization of the LacZ/EGFP dual-reporter at the cellular level in a 1-year-old Tg12084 rat. (**a**) β-Gal staining of a coronal section of a Tg12084 rat brain. Inset is a magnified region of the cortex depicting cortical layers I–VI. Scale bar = 1000 μM, inset = 500 μM. (**b**) EGFP positive cells expressing the transgene, (**c**) cells expressing the neuronal nuclear marker NeuN, and (**d**) merged image of (**b**) and (**c**) of layer III of the cortex. Scale bar (100 μM) in (**b**) also corresponds to (**c**) and (**d**). EGFP fluorescence (**e**–**g**), NeuN staining (**h**–**j**), and merged image (**k**–**m**) of the hippocampus (**e**, **h**, and **k**) and higher magnification of CA1 region (**f**, **i**, and **l**) identified by the solid boxes in (**e**), (**h**), and (**k**), and the CA3 region (**g**, **j**, and **m**), identified by the dashed boxes in (**e**), (**h**), and (**k**). Scale bar (500 μM) in (**e**) corresponds to (**h**) and (**k**). Scale bar (100 μM) in (**f**) corresponds to (**g**), (**i**), (**j**), (**l**), and (**m**)

Next, we studied LacZ and EGFP expression in adult Tg rats up to one year of age. We observed high EGFP fluorescence in the brains of 9-month-old Tg12084 and Tg12085 rats (Fig. 4a and b). Both lines demonstrated EGFP fluorescence in the cortex, corpus callosum, cerebellum, and brainstem (Fig. 4a and b). Tg12085 rats showed more widespread EGFP fluorescence that was higher in intensity in the brainstem and thalamic structures compared with Tg12084 animals. Conversely, Tg12084 rats had high EGFP fluorescence in the corpus callosum and forebrain (Fig. 4a). This region-specific EGFP fluorescence was maintained in one-year-old Tg12084 and Tg12085 animals (Fig. 4c and d). Tg12084 rats displayed higher EGFP fluorescence in the cortex, corpus callosum, and hippocampus (Fig. 4c). In contrast to the Tg12084 line, coronally sliced Tg12085 rat brain showed higher EGFP fluorescence in the thalamus and midbrain structures (Fig. 4d). To visualize the extent of RaPrnp-mediated expression in neural anatomical structures, we stained coronal sections of Tg12084 brain tissue with X-gal to measure LacZ activity in cells. While layer I lacked LacZ activity, we observed LacZ activity in cortical layers II–VI, with the highest reporter activity in layers II and III (Fig. 5a, inset). Furthermore, the nuclei of the CA1 layer and dentate gyrus showed stronger LacZ activity than the CA3 layer of the hippocampus, while the thalamus displayed sparse activity (Fig. 5a).

### RaPrnp drives transgene expression in rat neurons

**Fig. 6:**
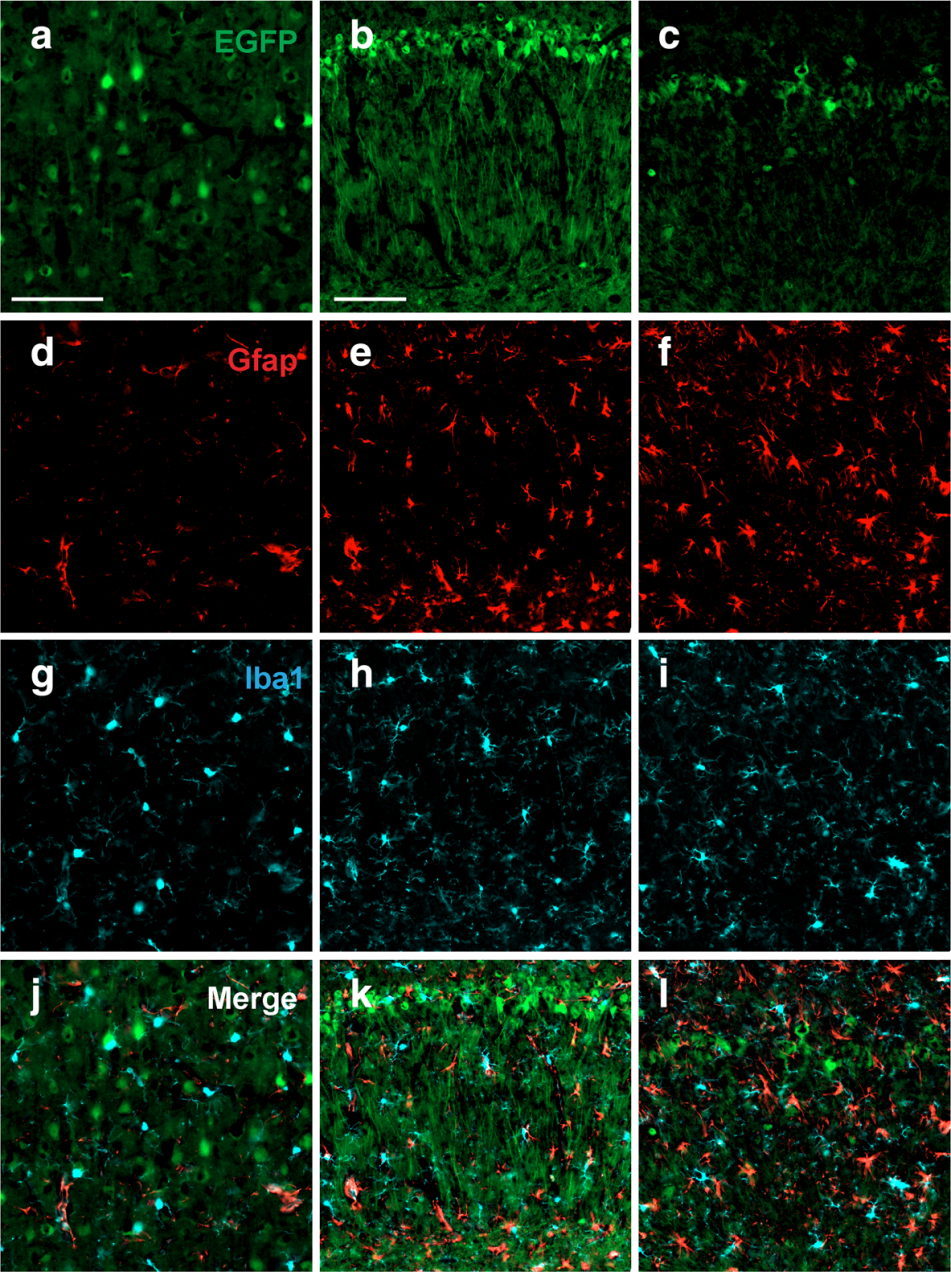
RaPrnp vector does not exhibit ectopic transgene expression in rat glia. Cortical layers IV and V (**a**, **d**, **g**, and **j**), CA1 region (**b**, **e**, **h**, and **k**), and CA3 region (**c**, **f**, **i**, and **l**). Immunofluorescence for EGFP driven by the RaPrnp vector (**a**–**c**), astroglial marker Gfap (**d**–**f**), and microglia marker Iba1 (**g**–**i**) shows no co-localization in Tg12084 rat brains in cortical or hippocampal regions. Merged images (**j**, **k**, and **l**). Scale bar (100 μM) in (**a**) corresponds to (**d**), (**g**), and (**j**). Scale bar (100 μM) in (**b**) corresponds to (**c**), (**e**), (**f**), (**h**), (**i**), (**k**), and (**l**)

To identify which CNS cell types were positive for RaPrnp-mediated expression, we performed immunofluorescence staining and confocal microscopy in Tg12084 rat brains to detect if neuronal or glia cell types expressed EGFP. Using this method, we observed EGFP to label cell bodies and axons of neurons that were also positive for the NeuN protein in the cortex (Fig. 5b–d). Furthermore, the hippocampus of Tg12084 rats showed widespread EGFP expression (Fig. 5e), whereas EGFP and NeuN were highly co-localized in neurons of the CA1 layer (Fig. 5l). Although EGFP expression in the CA3 layer was greatly reduced (Fig. 5g), it was apparent in neurons (Fig. 5m). Notably, in both regions, EGFP did not label all neurons but instead yielded mosaicism in neuronal populations. EGFP did not co-localize with either Iba1 or GFAP positive microglia or astrocytes, respectively, in the cortex (Fig. 6a, d, g, and j) or the hippocampal CA1 (Fig. 6b, e, h, and k) and CA3 (Fig. 6c, f, i, and l) layers, suggesting that RaPrnp-mediated expression primarily targets neuronal cell types.

### Using the RaPrnp vector to generate an accelerated rat scrapie model

We and others have shown that mice overexpressing mouse PrP^C^ have shorter incubation periods compared with WT mice infected with RML prions [2, 5, 23]. In addition, we demonstrated in a number of models that transgene expression level is inversely correlated to susceptibility to disease onset [15, 20, 22]. We therefore posited that genetically expressing higher levels of PrP^C^ would provide more substrate for PrP^Sc^ conversion and an accelerated prion disease phenotype in rats infected with rat-passaged RML (rat RML) prions.

**Fig. 7:**
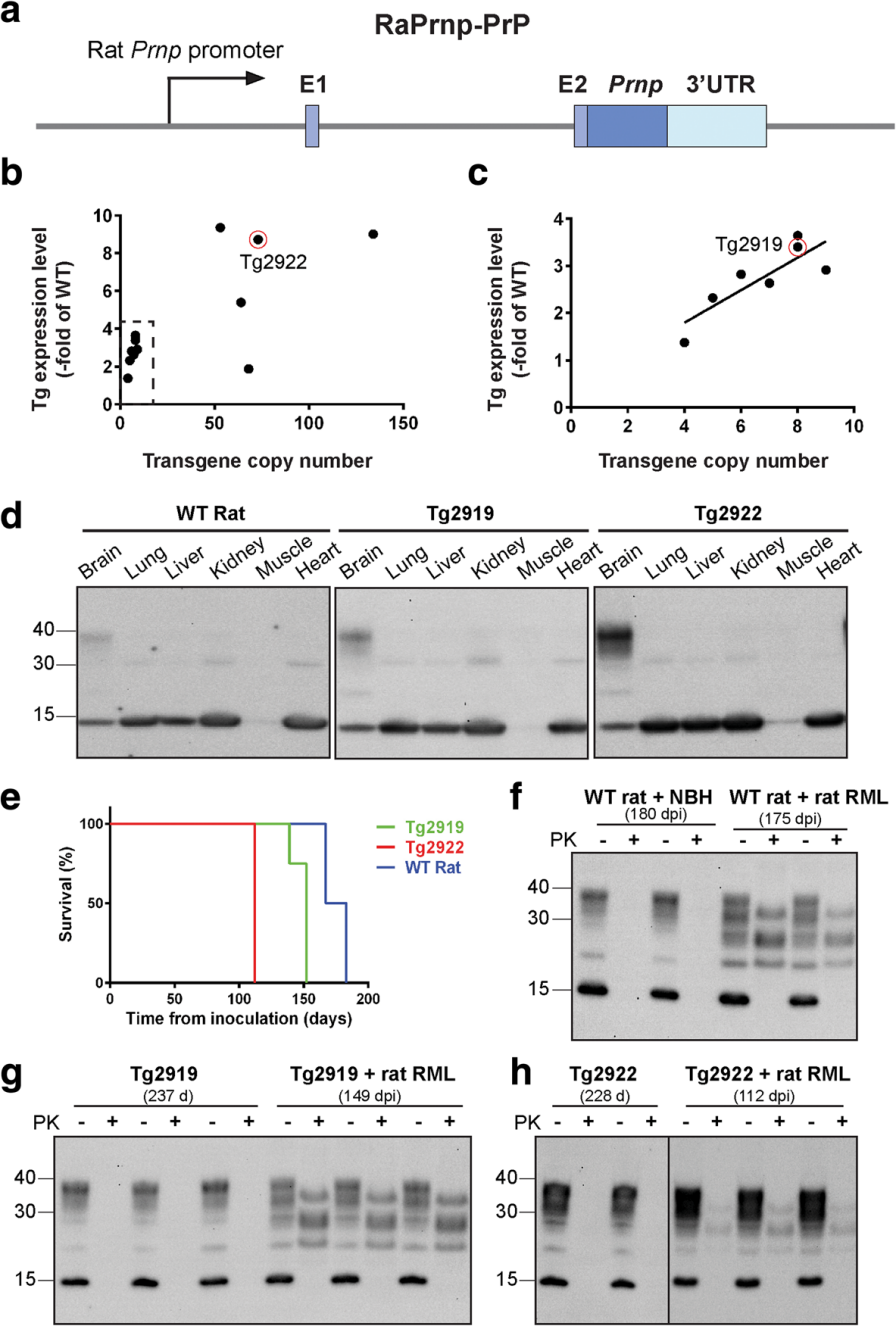
Rats overexpressing rat PrP show accelerated prion disease onset. (**a**) The rat *Prnp* ORF was restored in the RaPrnp vector by In-Fusion cloning. Schematic of the RaPrnp-PrP construct used to generate founder rat lines. (**b** and **c**) Comparing PrP expression level (y-axis, relative to WT PrP expression) to copy number (x-axis) in Tg(RaPrnp-PrP) founder rats. Graph in (**b**) compares all Tg(RaPrnp-PrP) founders, while the dashed boxed area is magnified to show the relationship between PrP expression and ≤10 copies of the RaPrnp-PrP transgene (**c**). Tg2922 and Tg2919 are indicated by red circles in (**b**) and (**c**), respectively. (**d**) Western blot showing rat PrP expression in multiple tissues from 4-month-old WT, Tg2919, and Tg2922 rats. (**e**) Kaplan–Meier plot demonstrates accelerated disease progression in rat RML-inoculated Tg2919 and Tg2922 rats compared with WT controls. (**f**) PK digestion studies of PrP in NBH or rat RML inoculated WT rats. (**g** and **h**) PK digestion studies of PrP in age-matched controls to Tg2919 and Tg2922 rats inoculated with rat RML. All western blotting experiments were done with an Anti-P antibody. Molecular weight is given in kilodaltons (kDa)

To overexpress rat PrP^C^ in the CNS, we PCR amplified the rat *Prnp* ORF with 15 bp homology arms and targeted insertion by In-Fusion cloning into an XhoI digested RaPrnp vector (Fig. 7a). This construct was microinjected into SD rat zygotes, and we achieved 81% viability, with 13% of implanted zygotes yielding live births (Table 1). Out of the 64 pups, we identified 15 potential founders (Table 1) by PCR genotyping. To determine whether transgene copy number was correlated with transgene expression level [19], we performed droplet digital PCR (ddPCR) using primer and probe sets targeting Exon I of the rat *Prnp* gene and western blotting to evaluate PrP protein levels (Fig. 7b and c; Online Resource, Additional file 1: Figure S1). Using this method, we detected copy numbers as low as 4 RaPrnp-PrP copies and a maximum of 132 copies in 12 Tg(RaPrnp-PrP) rats (Fig. 7b and c). For copy numbers greater than 10, we observed no correlation with RaPrnp-PrP copy number and PrP expression levels in the brain. In contrast, we observed a strong correlation with fewer than 10 RaPrnp-PrP copies on PrP expression levels (R^2^ = 0.70; Fig. 7c). Of the seven potential founders paired with WT rats, three did not produce Tg offspring, while two lines were poor breeders. For this study, we used two founder lines, Tg2919 and Tg2922, which had 7 and 71 RaPrnp-PrP copies, respectively (Fig. 7b and c). Tg2919 and Tg2922 lines had total PrP^C^ expression levels 4.4× and 9.7× of WT animals (Online Resource, Additional file 1: Figure S1).

To evaluate transgene-mediated tissue specificity, we performed western blotting and probed for total PrP expression in brain, lung, liver, kidney, muscle, and heart tissue extracts from WT, Tg2919, and Tg2922 rats (Fig. 7d). While we consistently found higher expression of glycosylated PrP in the range of 30–40 kDa in brain extracts from Tg2919 and Tg2922 rats compared with WT animals, we also observed a faint endogenous band around 30 kDa in lung, liver, kidney, and heart extracts with similar levels of expression in WT and Tg animals (Fig. 7d). The observation of a prominent ~15 kDa band was detectable in most tissues and was similarly expressed in WT and Tg animals (Fig. 7d).

To observe normal prion disease progression in WT rats, we inoculated rat RML prions into WT animals, which reached disease onset at 175 ± 3 days post inoculation (dpi) (Fig. 7e and Table 2). To test the effect of additional PrP in Tg rats on prion disease kinetics, we inoculated 2-month-old Tg2919 rats with rat RML prions. The animals reached disease onset at 149 ± 2 dpi (Fig. 7e and Table 2). To determine if we could further accelerate prion disease in rats, we inoculated Tg2922 animals, which express more than twice the level of PrP than Tg2919 rats. Rat RML-infected Tg2922 rats succumbed to rapid disease onset at 112 ± 0 dpi (Fig. 7e and Table 2). Interestingly, terminal Tg2922 rats had lower levels of PrP^Sc^ compared with rat RML-infected WT and Tg2919 animals (Fig. 7f–h).

**Table 2:** Comparison of scrapie disease in WT rats with Tg animals overexpressing PrP in the CNS

Line	Expression Level	Inoculum	Mean incubation period ± SEM (d)	Mean age at disease onset (d)	n/n_0_
WT rat	1×	rat RML	175 ± 3	224 ± 3	7/7
Tg2919	4.4×	None	N/A	>460	0/4
		rat RML	149 ± 2	210 ± 2	8/8
Tg2922	9.7×	None	N/A	>360	0/12
		rat RML	112 ± 0	198 ± 0	7/7

### Neuropathological characterization of accelerated prion disease in Tg rats

**Fig. 8:**
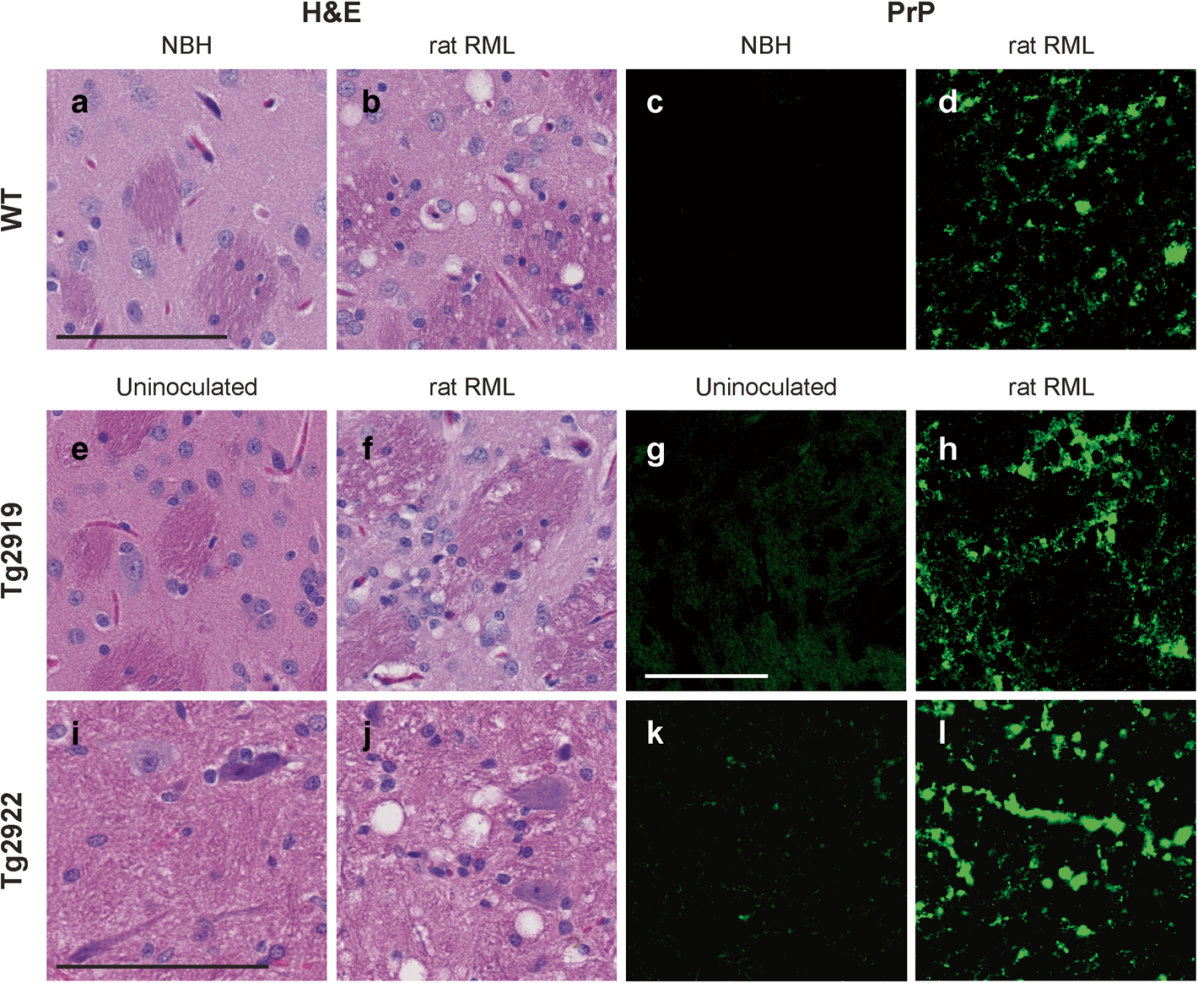
Neuropathology of WT and Tg rats. Neuropathological characterization of WT (**a**–**d**), Tg2919 (**e**–**h**), and Tg2922 (**i**–**l**) uninoculated rats or animals inoculated with NBH or rat RML. Vacuoles were mainly present at moderate to frequent densities in rats inoculated with rat RML; representative images of the striatum (**a**, **b**, **e**, and **f**) and brainstem (**i** and **j**). PrP deposition was absent in WT rats inoculated with NBH (**c**) but was moderate to frequent in rats inoculated with rat RML (**d**, **h**, and **l**). Only diffuse immunoreactivity was found in uninoculated Tg2919 animals (**g**). Some focal PrP deposits were seen in Tg2922 rats (**k**). Images from the striatum (**c**, **d**, **g**, and **h**) and brainstem (**k** and **l**). All scale bars = 100 μm. Scale bar in (**a**) corresponds to images in (**b**–**f**). Scale bar (**g**) corresponds to (**h**), and scale bar (**i**) to (**j**–**l**)

H&E staining was performed on WT, Tg2919, and Tg2922 RML-infected rat brain and compared with age-matched Tg control animals and WT rats inoculated with normal brain homogenate (NBH). Minimal vacuolization was observed in the brains of NBH-inoculated WT rats and age-matched uninoculated Tg2919 and Tg2922 controls (Fig. 8a, e, and i). In contrast, in both rat RML-infected WT and Tg2919 rats, frequent vacuolization was found within the hippocampus, striatum, and motor and sensory cortices (striatum depicted in Fig. 8b and f; Online Resource, Additional file 1: Figure S2). Conversely, Tg2922 rat brain showed the highest vacuolization within the brainstem (Fig. 8j), hypothalamus, and thalamus (Online Resource, Additional file 1: Figure S2). Hydrocephalus was apparent in all of the rats inoculated with rat RML and was most appreciable in the anterior horn of the lateral ventricles.

With respect to PrP immunofluorescence staining, all WT rats inoculated with NBH lacked PrP immunoreactivity following hydrolytic autoclaving (Fig. 8c). Notably, uninoculated Tg2919 animals occasionally had diffuse PrP staining in the striatum (Fig. 8g), while Tg2922 rats had some focal PrP immunoreactivity within the brainstem (Fig. 8k). In addition, both lines had some PrP immunoreactivity in the cingulate gyrus and hippocampus, which was likely a result of overexpression. WT and Tg2919 rats inoculated with rat RML prions had abundant PrP immunoreactivity, showing PrP deposits across numerous brain regions including the brainstem, cerebellum, hippocampus, thalamus, striatum, and motor and sensory cortices (representative photos of the striatum in Fig. 8d and h). PrP deposits in rat RML-infected Tg2922 rats were most severe within the brainstem (Fig. 8l) but were also present in the cerebellum, sensory cortices, and thalamus. No appreciable PrP deposits were noted in the motor cortices or striatum. Brains of prion-infected mice and rats are characterized by astrocytic gliosis; however, Tg2919 and Tg2922 rat lines had a high basal level of astrocytic gliosis, and, therefore, no upregulation was apparent in prion-infected rats.

## Discussion

Here, we report the RaPrnp vector, a novel Tg tool for the investigation of ND in Tg rats. Previously, our group developed the cos.Tet vector, which used the Syrian hamster *Prnp* genomic locus to drive the expression of a variety of *Prnp* transgenes in mice [16]. While this cloning vector was essential for understanding many of the fundamental properties of prion diseases in mammals, the large size of the vector (~43 kb) made it challenging for efficient cloning and transgenesis. Subsequently, the so-called “half-genomic PrP” expression vector (~12 kb), a fraction of the size of the cos.Tet vector, was used to rescue scrapie infectivity by overexpressing full-length or truncated MoPrP transgenes in *Prnp*-null mice infected with RML prions [5]. Furthermore, Borchelt and others created a vector termed MoPrP.Xho (~11 kb) [1], which has subsequently been used to derive a range of ND models in mice. While mice, rats, and hamsters are evolutionarily similar, mouse and hamster genetic tools may not encompass all the necessary genetic elements to confer strong and widespread rat-specific gene expression in the CNS. Based on our computational analysis of the *Prnp* locus in three rodent species (Fig. 1a), we identified regions that may contain essential regulatory elements in the rat *Prnp* gene for the RaPrnp vector.

While we were able to demonstrate pan-neuronal expression of LacZ/EGFP in adult staged rats from separate founder lines, the animals had different spatial and temporal activation of the RaPrnp vector. Expression in the Tg12085 line began at P10, whereas the Tg12084 line displayed gene expression as early as E13.5 in the developing rat CNS (Fig. 3), suggesting that the site of transgene integration may play a role in expression. The Tg12084 line might prove a powerful tool for neurodevelopmental studies where discrete populations of cells need to be carefully observed, microdissected, or collected via fluorescence-activated cell sorting. Moreover, because both Tg(RaPrnp-LacZ/EGFP) lines display robust in vivo EGFP fluorescence in young and aged adult brains, these lines may be useful for transplantation/grafting studies. While we observed some mosaic expression in Tg(RaPrnp-LacZ/EGFP) lines, expression was largely confined to neuronal populations, thus strengthening the use of this tool for genetically targeting rat neuronal circuitry for disease modeling. In addition, because the RaPrnp vector led to sustained LacZ/EGFP gene expression in older animals, it may be useful for long-term disease models.

To determine whether the new vector could be used to generate a model of ND in rats, we focused on PrP prion disease and generated several Tg(RaPrnp-PrP) potential founders. In general, higher copy numbers yielded variable PrP expression, possibly because high expression levels cause embryonic lethality, and thus only relatively lower-expressing rats survive. Conversely, we observed a good correlation between transgene copy number and PrP expression with 10 copies or fewer of RaPrnp-PrP. Focusing on lines Tg2919 and Tg2922 with different PrP expression levels, we found that most transgene expression was brain specific (Fig. 7d). This is in contrast with the MoPrP.Xho vector, which not only leads to expression in the brain but also to ectopic expression in the heart [1]. Brain-specific expression is advantageous, as it reduces unwanted off-target effects in other organs of these rats. Furthermore, by using the RaPrnp vector, we accelerated prion disease by ~15% and ~36% in Tg2919 and Tg2922 animals respectively, compared with WT controls (Fig. 7e and Table 2). These Tg rats also showed biochemical and pathological characteristics of prion disease. Interestingly, rat RML-infected Tg2922 animals showed disease onset at 112 ± 0 dpi but had less proteinase K–resistant PrP compared with Tg2919 rats (Fig. 7e, g, and h and Table 2). One explanation for this observation could be that the highly localized expression of rat PrP^C^ in Tg2922 rats leads to clinical signs before disease spread throughout the brain. This would then precede the higher accumulation of rat RML prions observed in infected Tg2919 and WT rats that express PrP at lower levels. This hypothesis is supported by apparent focal vacuolization and rat PrP^Sc^ immunoreactivity in infected Tg2922 animals. Due to this unexpected outcome of region-specific phenotypes of scrapie in the rat brain, these Tg rat lines may be a powerful new tool to evaluate CNS vulnerability in prion disease. Furthermore, while it is common for protein aggregation to occur under overexpressing conditions, we did observe some low level PrP protein aggregation in Tg2919 and Tg2922 aged control rats via immunostaining, although surplus levels of PrP^C^ did not cause any neurological phenotypes nor were detrimental to the life span of the animals.

Interestingly, Tg2919 and Tg2922 lines had PrP^C^ expression levels 4.4× and 9.7× (total of transgene and endogenous expression), respectively, to WT rats. Inoculating mouse Tg4053 and Tga20 lines, which express MoPrP at ~4–6× higher levels than those in WT mice, with RML prions yielded a > 50% reduction in incubation period compared with WT controls [2, 5]. This may indicate that mice and rats have different susceptibilities to scrapie infection. Also, the mechanisms of PrP^Sc^ propagation and clearance may be different between mice and rats. Whether delayed scrapie pathogenesis is due to more distant connections between neurons and/or neural anatomical regions remains to be determined in rats. Furthermore, while we cannot rule out that endogenous rat *Prnp* may influence the conversion of Tg rat PrP^C^ to PrP^Sc^, a *Prnp*(0/0) rat expressing PrP transgenes may address this question in the future.

Our findings suggest that elucidating modified phenotypes in the rat may lead to an improved rodent model to investigate ND. While altering prion disease in rats served as an important validation step for the RaPrnp vector, this new tool can be equally applied to modeling AD, PD, MSA, and the tauopathies in rats. This strategy is feasible as the RaPrnp vector is amendable for simple cloning and efficient transgenesis leading to high levels of expression throughout the rat brain. Because the rat offers many advantages to mice, including higher-order cognition, rat behavioral changes may be more prominent in future ND models. Also, rats have larger brains, making dissection of brain structures simpler for detailed transcriptome or proteomic studies of ND progression. Larger brains in rats may also provide better spatial resolution for microPET imaging compared with mice. Lastly, greater sample volumes of blood and CSF can be collected from rats making efficacy studies more advantageous in this animal model to investigate therapeutics for NDs. Novel tools such as the RaPrnp vector will allow investigators to refine and create new Tg rat models, ushering in a new era of ND modeling.

## Additional file

Additional file 1: This file contains an extended materials and methods section, supplementary references, Figures S1 and S2, Table S1, and Supplementary Information 1. (DOCX 7870 kb)

## References

[1] Borchelt DR, Davis J, Fischer M, Lee MK, Slunt HH, Ratovitsky T, Regard J, Copeland NG, Jenkins NA (1996). A vector for expressing foreign genes in the brains and hearts of transgenic mice. Genet Anal.

[2] Carlson GA, Ebeling C, Yang S-L, Telling G, Torchia M, Groth D, Westaway D, DeArmond SJ, Prusiner SB (1994). Prion isolate specified allotypic interactions between the cellular and scrapie prion proteins in congenic and transgenic mice. Proc Natl Acad Sci U S A.

[3] Cohen RM, Rezai-Zadeh K, Weitz TM, Rentsendorj A, Gate D, Spivak I, Bholat Y, Vasilevko V, Glabe CG (2013). A transgenic Alzheimer rat with plaques, tau pathology, behavioral impairment, oligomeric Aβ, and frank neuronal loss. J Neurosci.

[4] Filipiak WE, Saunders TL (2006). Advances in transgenic rat production. Transgenic Res.

[5] Fischer M, Rülicke T, Raeber A, Sailer A, Moser M, Oesch B, Brandner S, Aguzzi A, Weissmann C (1996). Prion protein (PrP) with amino-proximal deletions restoring susceptibility of PrP knockout mice to scrapie. EMBO J.

[6] Frankmann SP (1986). A technique for repeated sampling of CSF from the anesthetized rat. Physiol Behav.

[7] Herculano-Houzel S, Mota B, Lent R (2006). Cellular scaling rules for rodent brains. Proc Natl Acad Sci U S A.

[8] Hoesche C, Sauerwald A, Veh RW, Krippl B, Kilimann MW (1993). The 5′-flanking region of the rat synapsin I gene directs neuron-specific and developmentally regulated reporter gene expression in transgenic mice. J Biol Chem.

[9] Lee Y, Messing A, Su M, Brenner M (2008). GFAP promoter elements required for region-specific and astrocyte-specific expression. Glia.

[10] Leon WC, Canneva F, Partridge V, Allard S, Ferretti MT, DeWilde A, Vercauteren F, Atifeh R, Ducatenzeiler A (2010). A novel transgenic rat model with a full Alzheimer's-like amyloid pathology displays pre-plaque intracellular amyloid-beta-associated cognitive impairment. J Alzheimers Dis.

[11] Liu L, Duff K (2008) A technique for serial collection of cerebrospinal fluid from the cisterna magna in mouse. J Vis Exp:e960 10.3791/960 PMC2762909 19066529

[12] Nuber S, Harmuth F, Kohl Z, Adame A, Trejo M, Schonig K, Zimmermann F, Bauer C, Casadei N (2013). A progressive dopaminergic phenotype associated with neurotoxic conversion of alpha-synuclein in BAC-transgenic rats. Brain.

[13] Phinney AL, Horne P, Yang J, Janus C, Bergeron C, Westaway D (2003). Mouse models of Alzheimer's disease: the long and filamentous road. Neurol Res.

[14] Prusiner SB (2017) Prion diseases. Cold Spring Harbor Perspectives in Medicine. Cold Spring Harbor Laboratory Press, Cold Spring Harbor

[15] Prusiner SB, Scott M, Foster D, Pan K-M, Groth D, Mirenda C, Torchia M, Yang S-L, Serban D (1990). Transgenetic studies implicate interactions between homologous PrP isoforms in scrapie prion replication. Cell.

[16] Scott M, Foster D, Mirenda C, Serban D, Coufal F, Wälchli M, Torchia M, Groth D, Carlson G (1989). Transgenic mice expressing hamster prion protein produce species-specific scrapie infectivity and amyloid plaques. Cell.

[17] Scott MR, Köhler R, Foster D, Prusiner SB (1992). Chimeric prion protein expression in cultured cells and transgenic mice. Protein Sci.

[18] Steppan SJ, Adkins RM, Anderson J (2004). Phylogeny and divergence-date estimates of rapid radiations in muroid rodents based on multiple nuclear genes. Syst Biol.

[19] Stranger BE, Forrest MS, Dunning M, Ingle CE, Beazley C, Thorne N, Redon R, Bird CP, de Grassi A (2007). Relative impact of nucleotide and copy number variation on gene expression phenotypes. Science.

[20] Tamgüney G, Giles K, Bouzamondo-Bernstein E, Bosque PJ, Miller MW, Safar J, DeArmond SJ, Prusiner SB (2006). Transmission of elk and deer prions to transgenic mice. J Virol.

[21] von Hörsten S, Schmitt I, Nguyen HP, Holzmann C, Schmidt T, Walther T, Bader M, Pabst R, Kobbe P (2003). Transgenic rat model of Huntington's disease. Hum Mol Genet.

[22] Watts JC, Giles K, Stöhr J, Oehler A, Bhardwaj S, Grillo SK, Patel S, DeArmond SJ, Prusiner SB (2012). Spontaneous generation of rapidly transmissible prions in transgenic mice expressing wild-type bank vole prion protein. Proc Natl Acad Sci U S A.

[23] Westaway D, Mirenda CA, Foster D, Zebarjadian Y, Scott M, Torchia M, Yang S-L, Serban H, DeArmond SJ (1991). Paradoxical shortening of scrapie incubation times by expression of prion protein transgenes derived from long incubation period mice. Neuron.

