# Supplementary material for: A novel vector for transgenesis in the rat CNS
Source: Acta Neuropathol Commun. 2017 Nov 21;5:84. doi: 10.1186/s40478-017-0484-y (PMC5697436; doi:10.1186/s40478-017-0484-y)
Supplement: Additional file 1: — This file contains an extended materials and methods section, supplementary references, Figures S1 and S2, Table S1, and Supplementary Information 1. (DOCX 7870 kb) [file 40478_2017_484_MOESM1_ESM.docx]

ONLINE RESOURCE


Supplementary Material:
A novel vector for transgenesis in the rat CNS

Acta Neuropathologica Communications

T. Peter Lopez^1^, Kurt Giles^1,2^, Brittany N. Dugger^1,2^, Abby Oehler^1^, Carlo Condello^1,2^, Zuzana Krejciova^1^, Julian Castaneda^1^, George A. Carlson,^1,2^ and Stanley B. Prusiner^1,2,3*^

^1^Institute for Neurodegenerative Diseases, Weill Institute for Neurosciences, University of California, San Francisco, San Francisco, CA, 94158, USA; ^2^Department of Neurology, University of California, San Francisco, San Francisco, CA, 94158, USA; ^3^Department of Biochemistry and Biophysics, University of California, San Francisco, CA, 94158, USA

*Corresponding author: Stanley B. Prusiner, stanley.prusiner@ucsf.edu

# Materials and Methods

**Cell culture**

Rat neuroblastoma (B35; ATCC-CRL-2754), rat embryonic fibroblast (RAT2; ATCC-CRL-1764), mouse neuroblastoma (N2a), and NIH\3T3 (ATCC CRL-1658) cells were grown in complete media containing DMEM (Thermo Fisher Scientific, 11960044), 10% fetal bovine serum (FBS; Thermo Fisher Scientific, 26140079), 1× Glutamax (Thermo Fisher Scientific, 35050061), and 1× Penicillin-Streptomycin (Pen-Strep; Thermo Fisher Scientific, 15140148). Cath.-a-differentiated-5 (CAD5) cells [4], a gift from Charles Weissmann, were cultured in complete media containing Opti-MEM (Thermo Fisher Scientific, 31985070), 9% FBS, and 1× Pen-Strep. Confluent cells were passaged 1:10 in fresh complete media. Proliferating rat PC-12 cells (ATCC-CRL-1721) were grown in medium composed of DMEM (Corning, MT10013CVEA) supplemented with 10% heat inactivated horse serum (HIHS, Thermo Fisher Scientific, 26050088), 5% FBS, and 0.5% Pen-Strep. Cells were split 1:4 in complete media. For differentiation, a single cell suspension was plated onto Poly-L-Ornithine (Sigma, P3655) and 1:30 matrigel (BD Matrigel Matrix Growth Factor Reduced, BD Biosciences, 356230) pre-coated coverslips (NeuVitro, GG-18-1.5-pre) and cultured in DMEM supplemented with 1% HIHS, 0.5% Pen-Strep, and recombinant rat beta-NGF (R&D Systems, 556NG100) at a final concentration of 100 ng/ml. Differentiation was complete after 5–7 days.

**Animal husbandry**

Animals were maintained in an AAALAC-accredited facility in accordance with the *Guide for the Care and Use of Laboratory Animals.* All animal procedures were approved by the University of California, San Francisco, Institutional Animal Care and Use Committee. Rats were maintained in static wire-top polycarbonate cages using soft PAPERCHIP bedding (Chicago, IL) that was changed weekly. Sentinel serology and PCR-based infectious disease surveillance of the rat colony (Charles River Research Animal Diagnostic Services, Wilmington, MA) were negative for endo- and ecto-parasites and the following pathogens: SEND, PVM, SDAV, KRV, H-1, RPV, RMV, NS-1, REO, RTV, and MPUL. Rats were housed under ABSL-2 conditions in an air-conditioned room (10+ air changes hourly) at 22.5 ± 1.4 °C, relative humidity of 45% ± 14%, and a 12-hour light/dark cycle. Animals had free access to a Tekland diet from ENVIGO (Indianapolis, IN) and tap water. Rats were group housed unless an animal’s health status necessitated individual housing.

**Spatial and temporal characterization of RaPrnp-LacZ/EGFP expression in rats**

Tg(RaPrnp-LacZ/EGFP) males were mated to WT rat females, and a visible mating plug the following day marked embryonic day 0.5 (E0.5). Tg(RaPrnp-LacZ/EGFP) samples encompassing E9.5 through E18.5 embryos, neonatal, and adult brains were harvested and transferred to PBS, and EGFP fluorescence was captured with a modular stereo microscope (Leica, MZ10F). For detection of LacZ activity and EGFP expression, adult Tg rats were perfused with ice-cold 4% PFA (w/v) in 0.1 M phosphate buffer, pH = 7.4 [1]. Brains were collected, washed, and cryopreserved in sequential incubations in 20% and 30% sucrose solutions (w/v). Samples were embedded in OCT solution (VWR, 25608-930) and sectioned at 10–20 µM on a cryostat (Leica). For LacZ/X-gal staining [5], slides were washed 3 times, 5 minutes each in detergent rinse (0.1 M phosphate buffer, pH ~7.4, 0.02% Nonidet P-40, 0.01% sodium deoxycholate, and 2 mM MgCl_2_; all reagents were purchased from Sigma). Brains were then incubated in X-gal staining solution (detergent rinse buffer, 5 mM K_3_[Fe(CN)_6_], 5 mM K_4_[Fe(CN)_6_] · 3H_2_O and 1 mg/ml X-gal) and covered overnight at 37 °C. Samples were post-fixed, washed in detergent rinse, and mounted with coverslips. Immunofluorescence was performed on 20 µM cryostat sections using procedures described in the neuropathology methods with Gfap (Abcam, 4674) 1:500, EGFP (Abcam, 6556) 1:1000, NeuN (Millipore MAB377) 1:100, and Iba1 (Synaptic Systems, 234004) 1:1000. Appropriate Alexa Fluor secondary antibodies (Thermo Fisher Scientific) were used at a dilution of 1:500.

**Generation of EGFP heat maps**

Coarse slices of coronal or sagittal Tg(RaPrnp-LacZ/EGFP) adult rat brains were added to a dish containing PBS. EGFP fluorescence was captured using a modular stereo microscope (Leica, MZ10F). Images were then set to an 8-bit format with the threshold setting set to a minimum of 0 and a maximum of 255 and applied to a HeatMap Histogram plugin in ImageJ software.

**Intracerebral inoculation of rats with brain homogenates**

Rats were intracerebrally inoculated with 50 µl of 1% brain homogenate (w/v) in 5% BSA diluent with rat-passaged RML prions (a gift from Allen Herbst and Judd Aiken) [3] or normal rat brain homogenate (NBH) as a control. Terminal rats were euthanized, and the brains were collected and bisected sagittally along the midline. Half brains were fixed in formalin, while the other half was frozen for pathological and biochemical analysis, respectively.

**Neuropathological analysis**

For vacuolation lesion analysis, nine areas—the striatum, hippocampus, thalamus, hypothalamus, brainstem, corpus callosum, motor cortex, sensory cortex, and cerebellar white matter—were assessed as previously described [2]. Briefly, semi-quantitative scoring of spongiform degeneration was conducted on H&E sections. The scoring was as follows: 0 (no vacuolation), 1 (mild vacuolation), 2 (moderate vacuolation), or 3 (severe vacuolation). Areas were not scored if there was insufficient tissue due to folding, tearing, etc.

**Tissue homogenization**

Tissue was weighed and added to a sterile 2 ml microfuge tube along with 2.3 mm diameter stainless steel ball bearings (BioSpec Products, 11079123ss), and ice-cold PBS was added to make a 10% (w/v) homogenate. Tissue was homogenized with a Burton Precellys 24 instrument, with a setting of 6200 for 1 minute and 20 seconds per round. This was repeated a total of 4 times with a 5-minute incubation step on ice before each round of homogenization. 10% homogenates were diluted 1/10 and quantified with a bicinchoninic acid assay kit (Thermo Fisher Scientific, 23225).

**PK digestion and western blotting**

Samples were diluted to 1 mg/ml (total protein) in lysis buffer containing 0.5% (w/v) DOC and 0.5% (v/v) NP40 and digested with 20 µg/ml PK solution (Thermo Fisher Scientific, AM2548) for 1 hour at 37 °C. Reaction was stopped with 1 mM PMSF (Sigma) and incubated on ice followed by ultracentrifugation for 1 hour at 48,000 rpm at 4 °C. Supernatant was discarded, and the pellet was resuspended in 100 µl of 1× LDS Sample Buffer (Thermo Fisher Scientific, NP0007). Prepared samples were applied to a 4–12% Bis-Tris PAGE gel (Thermo Fisher Scientific, WG1402), transferred to PVDF, and probed with antibodies using standard western blotting protocols. Antibodies were used at the following dilutions: 1:5,000 Anti-PrP antibody (mouse P) and 1:10,000 Goat Anti-Mouse IgG (H+L) HRP conjugated (Jackson ImmunoResearch Laboratories, 115-035-003). Using Image Lab software (Bio-Rad), quantitation of PrP^C^ expression levels in founder animals was determined by subtracting a one-fold difference of endogenous WT rat PrP^C^.

# Supplementary References

1 Gage GJ, Kipke DR, Shain W (2012) Whole animal perfusion fixation for rodents. J Vis Exp: e3564

2 Giles K, Berry DB, Condello C, Dugger BN, Li Z, Oehler A, Bhardwaj S, Elepano M, Guan S, Silber BMet al (2016) Optimization of aryl amides that extend survival in prion-infected mice. J Pharmacol Exp Ther 358: 537–547

3 Herbst A, Ness A, Johnson CJ, McKenzie D, Aiken JM (2015) Transcriptomic responses to prion disease in rats. BMC Genomics 16: 682

4 Mahal SP, Baker CA, Demczyk CA, Smith EW, Julius C, Weissmann C (2007) Prion strain discrimination in cell culture: the cell panel assay. Proc Natl Acad Sci USA 104: 20908–20913

5 Peter Lopez T, Fan CM (2012) A transgenic Tbx6;CreERT2 line for inducible gene manipulation in the presomitic mesoderm. Genesis 50: 490–495

**
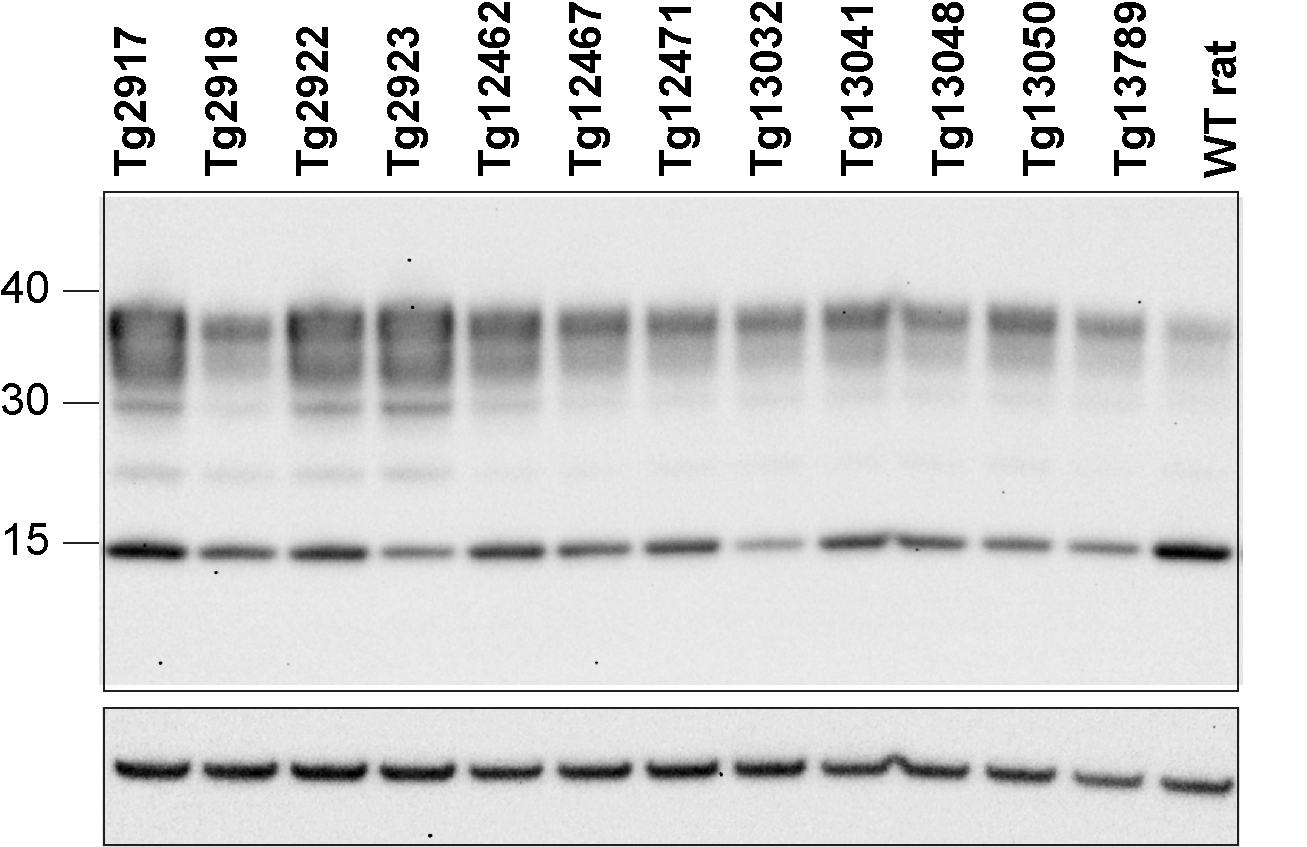
**

**Figure S1. Detection of PrP overexpression in Tg rats by western blot analysis.** The top western blot demonstrates PrP expression in Tg founders and a WT control brain; the bottom blot depicts an actin loading control. Molecular weight is given in kilodaltons (kDa).


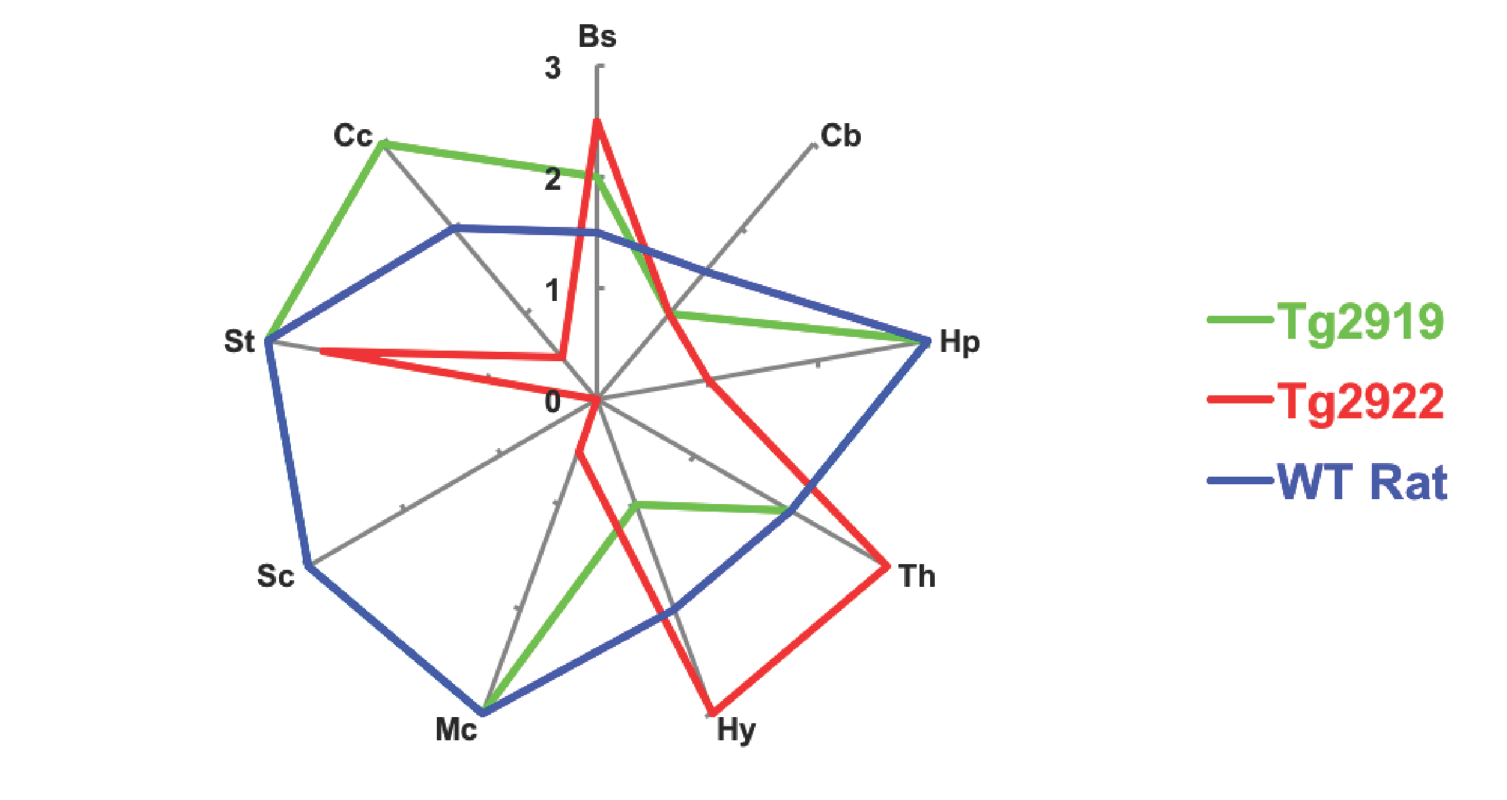


**Figure S2.** **Lesion profiling of WT and Tg rats infected with rat RML.** Radar plot showing median semi-quantitative vacuolation scores in WT and Tg rats infected with rat RML: WT rat (blue), Tg2919 (green), and Tg2922 (red). Brain regions are abbreviated as follows: brainstem (Bs), cerebellum (Cb), hippocampus (Hp), thalamus (Th), hypothalamus (Hy), motor cortex (Mc), sensory cortex (Sc), striatum (St), and corpus callosum (Cc).

**Table S1. Primers used in this study**

| **PCR amplified product** | **Forward primer** | **Reverse primer** |
| --- | --- | --- |
| In Fusion fragment: 15 bp pUC19 homology (H.) arm/RtPrnp promoter/Exon1/Intron1/Exon2/XhoI 15bp H. arm | 5'-TCGGTACCCGGGATCGCGGCCGCATGCACAAATACACAC-3' | 5'-ACAAGCAGGCTCGAGGGTACCGCTAAGAAGGCAGAAT-3' |
| In Fusion fragment: 15bp Exon2 H. arm/XhoI/3'UTR/2.2 kb downstream sequence/15bp pUC19 H. arm | 5'-CTCGAGCCTGCTTGTTCCTTCTCATTC-3' | 5'-CGACTCTAGAGGATCGCGGCCGCGAGAGCTCATCAGTGAAGTTGG-3' |
| In Fusion fragment: 15bp XhoI 15bp H. arm/LacZ/TA linker/15bp EGFP H. arm | 5'-TAGCGGTACCCTCGAATCCACCATGGGGCCCAA-3' | 5'-ATGTTAGCAGACTTCCTCTGCCCTCTCCGGAACCTTTTTGACACCAGACCAACTG-3' |
| In Fusion fragment: 15bp LacZ H. arm/T2A/EGFP/15 bp 3'UTR H. arm | 5'-GAGGGCAGAGGAAGTCTGCTAACATGCGGTGACGTCGAGGAGAATCCTGGCCCAATGGTGAGCAAGGGCGAG-3' | 5'-AACAAGCAGGCTCGATTACTTGTACAGCTCGTCC-3' |
| In Fusion fragment: 15bp RtPrnp H. arm/Rat Prnp ORF/15 bp RtPrnp H. arm | 5'-TAGCGGTACCCTCGAGCCGCCACCATGGCGAAC-3' | 5'-AACAAGCAGGCTCGATCATCCCACGATCAGGAAG-3' |
| pUC19 sequencing primer | 5'-GCAGATTGTACTGAGAGTGCACCA-3' |  |
| Intron I Forward sequencing primer | 5'-GATTGAACCCAGGGATAG-3' |  |
| 3'UTR Reverse sequencing primer | 5'-CCTGACTAGCCAGCAGTTAT-3' |  |
| 3' Prnp genomic locus Forward sequencing primer | 5'-TGCTGCCATGTTCTTGCCTTGATG-3' |  |
| RaPrnp Forward genotyping primer | 5'-CAACCCAACTGAAGTATTCTGCCTTC-3' |  |
| Rat Prnp ORF Reverse genotyping primer |  | 5'-TGCCACATGCTTGAGGTT-3' |
| LacZ-T2A-EGFP Reverse genotyping primer |  | 5'-GATAGGTTACGTTGGTGTAGATGG-3' |

**Supplementary Information 1. RaPrnp vector sequence**

pUC19 sequence

Not1 site

Rat *Prnp* promoter

Exon I

Intron I

Exon 2

XhoI

3’UTR

~2.2kb *Prnp* downstream sequence

GATCCTCTAGAGTCGACCTGCAGGCATGCAAGCTTGGCGTAATCATGGTCATAGCTGTTTCCTGTGTGAAATTGTTATCC

GCTCACAATTCCACACAACATACGAGCCGGAAGCATAAAGTGTAAAGCCTGGGGTGCCTAATGAGTGAGCTAACTCACAT

TAATTGCGTTGCGCTCACTGCCCGCTTTCCAGTCGGGAAACCTGTCGTGCCAGCTGCATTAATGAATCGGCCAACGCGCG

GGGAGAGGCGGTTTGCGTATTGGGCGCTCTTCCGCTTCCTCGCTCACTGACTCGCTGCGCTCGGTCGTTCGGCTGCGGCG

AGCGGTATCAGCTCACTCAAAGGCGGTAATACGGTTATCCACAGAATCAGGGGATAACGCAGGAAAGAACATGTGAGCAA

AAGGCCAGCAAAAGGCCAGGAACCGTAAAAAGGCCGCGTTGCTGGCGTTTTTCCATAGGCTCCGCCCCCCTGACGAGCAT

CACAAAAATCGACGCTCAAGTCAGAGGTGGCGAAACCCGACAGGACTATAAAGATACCAGGCGTTTCCCCCTGGAAGCTC

CCTCGTGCGCTCTCCTGTTCCGACCCTGCCGCTTACCGGATACCTGTCCGCCTTTCTCCCTTCGGGAAGCGTGGCGCTTT

CTCATAGCTCACGCTGTAGGTATCTCAGTTCGGTGTAGGTCGTTCGCTCCAAGCTGGGCTGTGTGCACGAACCCCCCGTT

CAGCCCGACCGCTGCGCCTTATCCGGTAACTATCGTCTTGAGTCCAACCCGGTAAGACACGACTTATCGCCACTGGCAGC

AGCCACTGGTAACAGGATTAGCAGAGCGAGGTATGTAGGCGGTGCTACAGAGTTCTTGAAGTGGTGGCCTAACTACGGCT

ACACTAGAAGAACAGTATTTGGTATCTGCGCTCTGCTGAAGCCAGTTACCTTCGGAAAAAGAGTTGGTAGCTCTTGATCC

GGCAAACAAACCACCGCTGGTAGCGGTGGTTTTTTTGTTTGCAAGCAGCAGATTACGCGCAGAAAAAAAGGATCTCAAGA

AGATCCTTTGATCTTTTCTACGGGGTCTGACGCTCAGTGGAACGAAAACTCACGTTAAGGGATTTTGGTCATGAGATTAT

CAAAAAGGATCTTCACCTAGATCCTTTTAAATTAAAAATGAAGTTTTAAATCAATCTAAAGTATATATGAGTAAACTTGG

TCTGACAGTTACCAATGCTTAATCAGTGAGGCACCTATCTCAGCGATCTGTCTATTTCGTTCATCCATAGTTGCCTGACT

CCCCGTCGTGTAGATAACTACGATACGGGAGGGCTTACCATCTGGCCCCAGTGCTGCAATGATACCGCGAGACCCACGCT

CACCGGCTCCAGATTTATCAGCAATAAACCAGCCAGCCGGAAGGGCCGAGCGCAGAAGTGGTCCTGCAACTTTATCCGCC

TCCATCCAGTCTATTAATTGTTGCCGGGAAGCTAGAGTAAGTAGTTCGCCAGTTAATAGTTTGCGCAACGTTGTTGCCAT

TGCTACAGGCATCGTGGTGTCACGCTCGTCGTTTGGTATGGCTTCATTCAGCTCCGGTTCCCAACGATCAAGGCGAGTTA

CATGATCCCCCATGTTGTGCAAAAAAGCGGTTAGCTCCTTCGGTCCTCCGATCGTTGTCAGAAGTAAGTTGGCCGCAGTG

TTATCACTCATGGTTATGGCAGCACTGCATAATTCTCTTACTGTCATGCCATCCGTAAGATGCTTTTCTGTGACTGGTGA

GTACTCAACCAAGTCATTCTGAGAATAGTGTATGCGGCGACCGAGTTGCTCTTGCCCGGCGTCAATACGGGATAATACCG

CGCCACATAGCAGAACTTTAAAAGTGCTCATCATTGGAAAACGTTCTTCGGGGCGAAAACTCTCAAGGATCTTACCGCTG

TTGAGATCCAGTTCGATGTAACCCACTCGTGCACCCAACTGATCTTCAGCATCTTTTACTTTCACCAGCGTTTCTGGGTG

AGCAAAAACAGGAAGGCAAAATGCCGCAAAAAAGGGAATAAGGGCGACACGGAAATGTTGAATACTCATACTCTTCCTTT

TTCAATATTATTGAAGCATTTATCAGGGTTATTGTCTCATGAGCGGATACATATTTGAATGTATTTAGAAAAATAAACAA

ATAGGGGTTCCGCGCACATTTCCCCGAAAAGTGCCACCTGACGTCTAAGAAACCATTATTATCATGACATTAACCTATAA

AAATAGGCGTATCACGAGGCCCTTTCGTCTCGCGCGTTTCGGTGATGACGGTGAAAACCTCTGACACATGCAGCTCCCGG

AGACGGTCACAGCTTGTCTGTAAGCGGATGCCGGGAGCAGACAAGCCCGTCAGGGCGCGTCAGCGGGTGTTGGCGGGTGT

CGGGGCTGGCTTAACTATGCGGCATCAGAGCAGATTGTACTGAGAGTGCACCATATGCGGTGTGAAATACCGCACAGATG

CGTAAGGAGAAAATACCGCATCAGGCGCCATTCGCCATTCAGGCTGCGCAACTGTTGGGAAGGGCGATCGGTGCGGGCCT

CTTCGCTATTACGCCAGCTGGCGAAAGGGGGATGTGCTGCAAGGCGATTAAGTTGGGTAACGCCAGGGTTTTCCCAGTCA

CGACGTTGTAAAACGACGGCCAGTGAATTCGAGCTCGGTACCCGGGATCGCGGCCGCATGCACAAATACACACACCCCAG

AAAGAAAGAGACCCCTCTCTCCCAGCTTGTTTTCAGTAGATACAGAGTGCTTGTAAAACATGGGGTATAAACTGAACTCT

GAGAGTAACTTAAATCAGTGTCTAACAAAGAAAGAGGAAAGAGACAAGGAAACTGAGAGCAGTGACCTGAAGATTGTTAG

GTAATCTCCACGCCTTGCCCCCTTGATGTCAGAATGCTTCCTCATTTGCCTCTTGAAATTCTTTAGAAGCCAAAATTTCA

CAATTAGTCTTTGTGGTGGTTTGAATATGCTTGCCCCATGGTAAGTGGCCCATGAAGTGTGTTCTTGTTGGAATAGGTGT

GGTCTTGTTGGAGAAAGGCTGCCACTGTGTAGGCAGGCTTTGCGGACTCCTGGTGCTCAAGCTCCTCCCAGTGCATGAGA

GCCCCCATTCCTGGCTGCCTGCAGAAGATAGTCTCCTGCTGCTGCCTTTGAAGCAAGATGTAGAACCCTCAGCTCCTCCA

GCACCATGTCTGCCTGGATGTGTCCCACCATGATGATAATGGACTGAACCTCTGAAACCGTAAGCCAGCCCCAACTAAAT

GTTTTCTTTCATAATAGTTGCCTTGGTCATGTTGTCTGTTCATGGCAATAAAACACTAACTAAGACAGTCTTAAATCAAT

CAAAAGACCTTTAAGTATTGAACAAACACCATTTTCATGTATCAAGTTGGCAATGATTAGAAAACAACTGTAGTTCTACA

TCAGAAGACTCAGGGAACCTTTTGGAGAAATATACTCATCCAAGCATGTTGGCTTCTTGATTCCAAAGCCAAGACACCTG

CCAAGCCTTTCCATGCCATGGATGGATCCCAGCATGGCATCTGGCTGCAGGGATGTGTGCATGGGGCAACCACTGGAAGG

CACTGAAACAACTGAAACAGCATCACAGAAGATATTGCAACCCAGGGAGAGACTAGTTCAAAGCTCAGAACTATACCTGG

TTAGGTGCAGAAAAGACGAGAGGACTAAAACCAGGAATATTTTAAAATATTTTTATTGATTTCATGTTCATAGATGTTTT

GTCTGAAAGTCTGTCTGTGTACCACATGCATGGCTGGCTCCTGCAGAGGCCAGAAGAGAGCATCAGATCCCCTAGAGCTG

GAGTTTCAGAAATTTGTAAGCTACTACATGGGTGCTGGGAACCGAACCCAGAACATCTGGAAGAGCAGCCAGTGTTCTTA

ACTACTGAGCTAACTGCTCAAGTCCCCCCATGAATGTTTTTCTTTATTTTTGTCTGTATTTTCCAATGTACTTACTGGAG

ATATACAACTTCTGTCATACATAACAAATAATTGAAGAAATGAAGCGAGAGGAGCAGCTGTTTAAATGCTGCCTGGGGCT

TGGCTGCCCCTGGCCTGGGTGTCCCGGTGCCAGCTGTCATGTACTGCGTCATAATAAGTTTCATTTGGTGCTGAAGGGAT

GTCTCAGCCTTTAAAGGCTAAGGCTCACAACCAAAATAAGTTGCATTTTCTTTGCACCAGGCTGAAGGGGGCTCTGGAGC

TTGCTGTCAATGTTACGTAACAGTCTAACTTCAAATTCAAACCATCTGGACATGCTAACCAAAGAACTAAACCACATGTT

GTAGTGCTTCTCTACCTCTACCATTCAAAGCTTTCAGACTTTGTGGAAATAACACCGAATACCACCAGGAGCCACAGAAA

CCATTTGCCTTAGTCCACTTCTGACACTATGACAGAATGTCACAGGCTCATTAAGAACCAGGGTGCCAACAGGCTTGGTG

TTGGTTTTAGGAGAGGCCTGTTTTACTGCTGTGTCCTCTGGGGATGGTGAGACCGCTGGATCCCATAACAAGGCAGAACA

TGGAGGGCATTAGGAAAAGCCGGGTGTGTGAGTTATTGTTCTGATTCAGTTCCTGACAAAAAGGACTTAAGGGAGAAAAC

TCGGGTAACAGGGAGTCCTTACAAGGAAGGCATGGCTGCGTTGACAGCAAGCATGTGGGTCAGGAGGGGGGAACTTAGAC

CAGCACCAGAGAAGTCTTCAAGGTCCACCTCAGTAAAGGGATGGCTCAGCCTTTAAAGGATAGGTCCACAACCAAAGTAA

GTTTTGTTTGCACCAGGCAGAAGGGGTTCTGAAACTTGCTGACCATATTATGTAACACTCTAACTTCAAATTTGGTATAG

GGCTCATACACCAAAGGTTCTATAAACCCCAGAACAGCACCACTCCCTGGAAACAAGCATCCACACACAAAAGCCTATGG

GGCACTTTATATCCAAACCATAAAGCCATGTTTGAAACTTCCTCTACAAGGGCCTGAATCCCATTTATGACGGGAAGGAG

GCCCCACTTCATAACAACACCCCACTGGTGACATTTGAAGGGGACACATTCAAGCCGTAACACTGTCTTTTGCCTACTAG

GGTTAAATTGTGCCATCTCGTCATTTCTAAGAAGACAGAAGTTTTCAAACCTGGGCTTTGAGCCACAGGTGTAATAGCCT

ACACCACCTACTTTCAGGCAACTTGCTCCTCCCCCAAGATTAGACCCCAATAGGCCCAGGGTCTGGAATGCGTCTAACAC

AGATGTAGGCTCTGCCTTGGCTTGAGTCCTCATGTCCCTCCACCATGTTCTCACTCTTTAGCTTCCCATTTCAAGGTGCC

TTTCCTTAGCAGAAAAAAATCAGAAGCATAAAGCATGCCCCTGGGGTCGCCAGTCTTCAGAGGGAATTTTGTGATGGTCT

CCTCAGAGCACGTTTGTCAAGTCTAGTTCGCCTCATTTTCTTTATTTAATGAAAGAAAAATAATGCGGTGTTGCAAATTA

GCTTTGGTAATAGCTCCAACCATTGCCGTGTTCACAGTCTCATTCTGCCGCTCAAAACAGCAAACCTGCCACACTCCCTG

TGTGTAACTCAGAACAGCCACTAAACAGCAGAAAGAGGTTTGACTCCTCTCGGCCTTTCCACAAACCCACCAGCCACATG

CTTATTTAAATTATGTCCTTTCTCCCAACAATGACTTCCCAAATGCTAGGTTGGCACACTTATACCACCATGCCCAGCCT

TGCATGTCAATAATAACCAGGTAGGTTATTATTATAACCCAGTAGCTGCCAAGCAGCCAGTCCCCTTTCCTTTCTAGAGA

CCTCCCAACTCCCATCCAAAAAGCTTTTAAAGCCACTTCCTGGTGGGAAGAGAGCAGTCAGCCAGTAGGTATTTGATTCT

TAGAGGAAAAAGCTGATATCTTTTAACCAAGCCTTTCAGAGTCCCCCTGTGGGAGAGGCCAGATGGAGGTGAGGGGAAGG

CTGGTCCCTTCCCTAAGTGAATCTACCCACTCCTCTCCTGCGTCCCCTGCCATCTCTGACAGCAAGTGGAGGGCCTCTGA

GAAATTCAAAGATGCGGTCACCCTGATCACAATGAGTACTCTCTGAGGCAGGAAGGCAAGGATCTGAAAGACGGTCAGTG

TATTCCTTAAGAACACAGAGTAAATGTTAAGCAGCCACCAGCACCACTCATTTCTAACAGGCTGAAATCTGGGTCATCTC

AGCCAAAGGCTCTGCTCCAGCCCTCTCATGCATGTCCTCTTCCCAAACAGCTGTCAAGAGCGTACCCACATATGTACACA

CGCACACACACACACACCTATTTTATCATAACTATAATCCTCTGGCTCCATGACTACTTCATAACAACTTTAATCCTCCT

GGCTCCAATACTCTCTTACCACCCTCTCCTCCTGGATCCTAATACTGGACACAAATATTTAATCCAAATCCAATTTTGTG

TTTGTCAATAATCTTCAGTGTCTCCCTCTCCTCACAGGGACAGGAGATTACATTTCCTTGTGGTTTATGACCCCTTCTGT

CCCAGTACTGGTAGCAATTTATATACATGGCATGGGAATATGTTATATGTGTATGGGGCTGTGTGCACATATATATGAGG

GCACATGTCATCCTATGTGTGGAGGCCAGAGGTCAATGTCACGTCTTCCCCAATCACTGTCCAGTGGTCCCTGGATTCCA

AACTCAGGTCCTCATGCTTGGGAACCAACCCAGCGCCCTGATTCCTAACCCCCTTCTCTTTCAAACAAGGTCTCATTATG

TTGCCTGGGTCAGCCTTGAACTTGAGTCTCCTGCCTCGACTTCTCTGATTGCAGGTTTTCACCTGTCCAAGTCAGCAGGC

ATCTTGAACAAGAGCATCATTTCCCTTAAGCTGCTTCAGGCGGTGTTCATGGGGGCTCTTAAATGTAGTGTACTTTTCCT

TTGGACACAGTAAAAGCAAAACAAGATTAAACGAGCTGGACACGGTGGTACATGTCAATAATCTGAGAACTCAGGAAGCT

GAGGCAGAAGCGTTGCTTTGAGTGTGAGGTCAGCTTGAACTACTTAGAGGGTAGAACAGGCCAACTTGGGCTAAGAGACT

CTGTCTCCAAACATAAAGAAAAAAGGGAGGGAGAGAAAAAGAAAGGAAAGGAAAGAAGGGAGAGAGTAGGACATCTAAGA

GCCTGAATGTTGGTGGTGTTGACCAGCCAGACTGAATTTAAAATGGGTAATGTTGAAAAAGGCAAGGAGATTTTTGTTCA

GAAGTGTGCCCAGTGTCACACTGTGGAGAAGAGAGGCAAACATGAGACTGGACCAAACCTCCGTGGTCTGTTTGGGTGGA

AGACAGGCCAGGCTGCTGAATTCTCTTACACAGACACCAACAAGAACAAAGGCATCACCTGAGGAGAGGATACCTTGATG

GAGTATTTGGAGAATCCCAAAAAGTACATCTCTGGAACAAAAATGATCTTCACTGGAATTAAGAAGGGAGAAAGGGCAGA

CCTAATAGCTTATCTTTAAAAGGCTGCTAATGAGTAATTCTACTGTCTTATTTATTACAAAACAAATGTCGTATGGCTTT

TAATGTATACCATAATTTAATTCATACACCAAATTCAGATCATGAATGGCTAACAATGTTTTTGTTGGACAGCCTTGATT

TAAGTAAAACTGACCTGTCATAGGTGGACATGATCTCTTTTAAAGTAACACTTCCAATTGAATACATGCTATCACTGCTC

TCCCTTTCTCAAGATAAGACTGGACTTAATTAGTAATGTTTTACTTTCCATAAATATGTGGCTGTCACCTCAAACCTATT

GAGTGTTTTTATACTTAGATCTGTATAACTGGGTCTATGAATATGTTTAAACACTGGGAAAATTCTATCGCTGTCTCAGA

AACAAGAAGACTCACCCGTGCTTCAGTGTGTGTTCACTGGCCTCTGACAGGCAATGCTAAACACCAGGAAGGAACTATTC

TTGACATTGCTGTTTTAATTAGAATTCCCTGGGGCTGGGGATTTAGCTCAGTGGTAGAGCGCTTACCTAGAAAGTGCAAG

GCCCTGGGTTCGGTCCCCAGCTCCGAAAAAAAAGAACCAAAAAAAAAAAAAAGAATTCCCTACATCAGTGATGCTGCCTT

TCACCACTGAAAGGCATTTACGGTGGTTTATGTATGATATCAAATAAAGAGTATTTAACACTTCTTTATAGTTGAAAAAG

AAAGGAAAGAAGGGAATCAACCGAGAATGATAAACCAACATTCAATGGCCAATATACTTTCTAAGCCTCTAATTCTTTTA

TAGTTTATGGGGAAATGTCAAAAATCTTCCTCTTTACCAATTTCTTGTTACCAAAGTTCCACGATGGCTTTTTCTTTCCG

TTAGGTAACCTTTCATTTTCTCGACTACCCATTATGTAACGGGAGCGCTGGGTTCTGGATCAGTCTTCCATTAAAGATGA

CTTTTATAGTCTGTGAGCGTCGTCACAGAGTGCTGACACTGGGGTGGGGAGGGGAGTACGGGGGGAGGGGGTTAAACAGA

TAACAAGCATTTAAGCCAGTACGGAGCGGTGACTCATCCCACCGCGAGAAGCCATTGGTGAGCATCACGCTCCGCCCCTC

GCCCCGCCCAGCCCCCGGCCTGTCGGGTCCCTCACCACGCCCCGCTCCCCCGCGTTGTCAGAGCAGCAGACGGAGTCTGA

GCGTCGCGTCGGTGGCAGGTAAGCGGGCTGCTGAAGCCAGGCGTCAGCGAGCATTCAGCCTTCCTCCCGTCGACAAGCTC

GGCTTACTGTGCCTCTCCGGGACTTGAGGCCGCGGGGCTGGGACTGGGGTTGAGCTTGGCTAGGAGGTGGCTGTGCACCC

GCTGCTGCGCGACTCCTGGAGGGACCGAATCCCAGGGCAGCGAGGCCGGGAGCCGAGCCTGATTCACAGCTCAACATCGC

TGTGGGGGATGGGGGGTTGGGGGGGTGGCATCTTTTAACTGCCCTGTGCTGTTTTCTTCTCTCGTTGTAATAGCTACAGC

GAACATAATTTCACCCCGTGATTCCACCACGGTCTCATCCGTCCTCAGCACCACACTCATTGCTCCCCTTGCTCAGTTTC

ATACTCAGCGCAGCCGTTCGCCTTCACTGCCCTGCCTAGGCGTTTTCATGGTTGTCTTATATTCTTTTACTTTGAATATC

GTGGTTTAATAGCAGTTGCCGGTGTGCTAAATTCCTCATTTCCTTAAGAGAAACTCCTGGGAGGATGGAATTAAAGACGT

TGCAAATTTAATTATACCACAAACAGGAATCAAAATTTTGCATTAAAATGCCAGACATCTTGAAAAATTTAACTATTCAA

TAAAAAAAAAAAAAGGAACTACTTTACCTACACACACATCCGAGTGCTTCAAAGAGTCCAAGGAAATAGAAAGCTAAGGG

ATGATTTGGGTTGTATTTGAATCTGACACGAGCTTTCCATATTATTTATAGCAGGGACTGAAGGATGAGTCATTTTCTGA

ATAAGATGCAAATTAAAGCAAGTTTGTTGTCTTTACATCGATTAAACAGACAGAGATGATGACAGCAGCAACCCTAACCT

AGAGGTTGTCTGAAACCACCGTGTTCAAGTTTGGGGAGCAGGTGGCCCTCCTTAAGAGCTCGATTGATTGCTTTACAACC

AACGTTATGACTTGGCATTGCCTGGGGTTCCTTTTATTTATTCCTTTCTTTAAAAGACTACTATCTATTTTATGAGCATG

AGTGTTTCGCTCCACAGAAGCATGTATACAAGCCTGGTTCTGCGGAGGTCAGAAGAGACAGGGTGTTGGAAGCCCTGGAA

CTAGAGCTAGGGATGATTCTGTGAGCCCCTGCCACAGGGGAGCTCAGAACCCCAATCCAGGCTGTCTGGAAGAGCAGCCA

GAGCTCTTAACTACCGAACACCCCCCCCCCATCCCCTCTCATTCACATTTAGAAAGGAGAAAACTGCTACCCATGTCTGG

CATTTATTTCAGAGATTAACTGTGCAAAACTCGATGTTGAAAGTATACTATTCTGTTTCCCATTCACACTTAGTTGACAG

TGTAAGTCAGTAAGGGCTTTTGTTTGTTTGTTTGGTTGGTTGGTTCCTGGGTTAGTCTGGAGTGTGCTTGTTTGAGAGCT

CAATAACAGGCTTTCAATATGGATATGTAGCTGGAATTCGCTATGTAGACCAGGCAGGCCTCAAATTTGTGGCAATCCTC

CCTGTGATTCCCCAGAATGCCCTGGTACAGGCATAAGCCACTGTGCCCAGCCGTAAAACAATCTGGTGAGGTATTATTAG

TTGCATGCTGTGACCCAGAAACCCCACTTCTGGCAATTCACCTGCCGTGGTGGAACCAACAAAGGGCTAGGGGAGCCATA

TGGCCAACAGTTACAGAAAATTAGATCCAAGGGAAAAGCAACCTAAATGTTTAACAGGCGAGCAGCTAAGAAACTGACAG

GCTGGTGAGGGAGCTGTAGCAATCCCGAAGAACACTCTTCATTTTAGACTCCATGTATCCCTGGGAAAAACAGAGTCAAA

GTACAGGTTAGGAGACCGGGACTCCTCTGGACCCATGCTGTCCTCTGAAAAGCCCAGAAGAGCTATAATGAAAGAGCTCA

GAAGATGTCTGATCTTGGCTTTCTTTATGTTTGTTGCTGTATTGTTTCCACTAACAAACAACTAAAAAAAAAAAAAAAAG

TTCACAGGCTTCTTTCCTTAAAATACTGGGGATTGAACCCAGGGATAGTTTTTTAGTGTCTAAATTAACATGACCATGCC

CTGTTTGCCTTTTTGGAGTATGTTTGAATCTGCCCTTATTTCCATTCTCAAATACTGCTCCATTTTATATGACTATTTAG

TTTTGGCTTGATAATTTGCATATGAGATTAGATCATCTTTCAGTTCTCAGACTTATTTATCAATTCTAGTTTTTCTTTTT

GTTGTTTTAAAGGACTCCTGAATATATTTCAAAACTGAACCATTTCAACCCAACTGAAGTATTCTGCCTTCTTAGCGGTA

CCCTCGAGCCTGCTTGTTCCTTCTCATTCTCGTGGTCTAGGCTGGGGGAGGGGTTACCCACCTGTAGCTCTTTCAATTGA

GGTGGTGTCTCATTCTTGCTTCTCTTTGTCCCCCATAGGCTAATACCCTTGGCAGTGATGGGTCTGGGGAAATGTACAGT

AGACCAGATGCTATTCGCTTCAGCGTCCTTTGATTGAGTCCATCATGGGCCAGGGTTAACACCAGGCCAGTAAGAATATA

ACACCAAATAACTGCTGGCTAGTCAGGGCTTTGTTTTGGTCTACTGAGTAAATACTGTGTAACCCCTGAATTGTACCCAG

AGGACATGGTGACAGAGACACACATAACTTAGTATAGGCAAAGGGTTCTATAGCCAAAGAAGCCACTGTGTGGGCATGGC

ACCCTGGAAAACAGCCTCCCGCCTGGGATATCTAGAGCATCCACATGTGGAATTCTTTCTTTTCTAACATAAACCATAGC

TGATTGAAGGCAACAAGAAAAAGAATCAAATTATCCTACTGACATTGAAAGCAAACTGTGTTCATTCCCTAGGCGCTGGA

ATGATTTTTAGCCTTGGATTAAACCAGGAGATTTTGACTCTGAGGAGAACCAGCAGTACAAAAGCATGGTCTCCTGTGAT

GGGAGAGATGGTGAAGGGACAAAGGCAAGACCCCTGCGTTTCTTCATTTCTGTCTCATAATTATCAAGAGCTAGAATTAG

GTCGTGCCCTAAGTTTCTGTACTCGTATTTGAACTGGACAACAAAGAGACAATCTACAAATTCTCTTGGGCTGCAGAGGA

GAGAAATAGGCTCCATTCCAAAGTGGAAAGAGAAATTCTGCTAGCATTGTCTAAGTAAGGCTAACTTTTCCTTAAATCGC

TTTGTATTTCCCCCAGCAGACATCACAACCCTGTGATCGGTTCAGCCTGCACCGCGGGTGTTCTGTGTAGAATATATAAA

TATAACTTCAAGCTTAGGCCTTCTATTTTAAAGCATCTGAAGTGTGGAACGCACTGGCCGTTCTGTGCAGTACTAAGTGT

GACCCTTGGGCTTTCAATGTGCACTCGGTTCCGTATGATTCCAAAGTAGAGCCCTAGCTGGTCTTCGAATCTGCATGTAC

TTCACGTTTTCTATATTTGTAACTTCGCATGTATTTGTTTTGTCATATAAAAAGTTTATAAATGTTTGCTATCTGACTGA

CATTAAATAGAAGCTATGATGAGCACGTGTGGGGTTTTTCTCCTTCAATGCTCCTGGCCCTGTGTTTGTCACAAGGGTGG

CTTGGGCTCATCTGAGCCTGGGAGTGGCTCATTTCACACCCTGTCCCTGATGGGCCTCTGATAAGGAGTCTTCTCATTTC

CACTGACTTTGCATTTTCCCTTAAGTTGTACTGAGTCCTGCAGAAACACAGTAGTACTCCCTGGGGAGGGAGGGAAGACT

GGGGAGTAGGGCTCCCCCACTCCAGAAGAGGTTTTTCTGTTTTTTGTTTTTTTCACTGTGAATCAGTGGTTTTGCCTACA

TATATGACTGTTCAAAAGATCCCCTGGAACTGAAGTTATGAATGGTTGTGAGCCACTGTGGGGTTTGGGACTTGAACCAG

GTACTCTGAAAGAACAGCCACTGGCCTTAACCACTGAGCTACTGCTTCAGCTCCAGGGAGTTTGTTTATTCTGAATTCAG

ATTCATTTTTCTCTCTCCTCCCTCCCTCCCTTTCTCCTCCTTCCCTCCCTTCCTCCTCCCTCCCTCCCTCCCTCCCCATT

TGTTTGGTCCATGGTGTGGTGATTGAGCCCAGGCCCTCAGACATACTGAGCTACACCTCAATCTGTAATTTTGAAATCAC

GTTAGTTTTTCTCTGGACTTGAACTCAAAGCAGTTCTCCTGCCCCAGTCTCCCTTGTGTTGGGGTTATAAAGGTGAACTC

CCATGCCCGGCTTAGACATAAATGTACTTTTTCTTTTGAGACAGGAATATCTATCTCTACAGAGCCCTGGCTGTCCCGGA

ACTCACTATGTCAAACAGGCTGGCCTCGAACTCACAGAGATCTGCCTGCCTCTGCTTCCTGAGTGCTGGGATTACAGGTG

TGCACATCTTAGAAATAGATTTTCTCGATTAAAATAAAAACTTAAGCTCCGAAGTGTCTGGTTTAAGCCCTCATAACTCA

GGCTTTGCAATTTCAGGTTTAACCTTTTGGCCCAAAAGGCCACACTTGCAATTCACTCTGAATACCTGTGTCTATTGCAA

GGAAAGGCCCTGGCTCACTGTTCAGGTGTCCGTTGTGGGGAGGCACCAGTGCAGCCATGTGATTGCTCACCGGGTAGGAA

TTTTCCTGAGCAGCTGCCATGTACAAATGCATAAAACCTGAAGTGGTGATGGAAACCACAATGTTCCATCCCACAGATGC

TCCAGCTGTGATAGCGCCACCTGCAGGCCTGTGAAGGTCACCCAGCCCTGCTGGGGAAAATCCCACTAGGAGAATTAAAG

CAGTCCTGATAGTCATGGGCCGTGTAAGAGAGCTCACAGGGGGCAACTCCACCAAGCAGGAGAGAGGGTAAGTTCTACAG

TGTGAAGGCCATGAAGCTCAGCCCTAGTCCTGAGGTTCGCCATTAGTGAGAAATTTTAGTTAATCAGGGTGGGAACCATC

CCACCAATGCAGGCTTCTCGAACTCTCACCAAGGCTAGTTATACGCAAGGAGACTGCAGACCTGAGATGAGCTCTATACT

TCCCAGAGTCTGTGAGGTCTGCAAGTTCCCAGGTGATAAATGACTGCTGATCTGAAGACCAGAAGCTGAAAACCAAGGTT

GGAGTCACCGATACTCCTCAAGCCTTGGACAAGCATTGGTGAAGTCAATGAGGGAGGGTGCAGAACCTGGCAAACATGTC

TTAGGCTCTCTTGTTTCTTTTGTTTGTGGGCGTGGTTTATGATAGTGTGACGGTTTGCATATGCTTGACCCAGGGAGTGG

CACTATTAGGAGGTGTGGCCTTGTTGGAGGAAGTGTGTCACTGTGGGGGTGGGCTTGGAGACCCTCCTCCTAGCTGCCTG

AGGATACTCAGTCAGTTCCTGGCTTCCTTCAGATGAAGATGTAGAACTCTCAGCTCCTCCTGCACCGTGCCTGCCTGGAT

GCTGCCATGTTCTTGCCTTGATGATAAAGGACTGAACCTCTGAACCTGTAAGCCAGCCCTAATTAAATGTTGTCCTTTAT

AAAACTTACATTGGTCATGGTGTCTGTTCACAGCAATAAAACCCTAACTAAGGCAGATAGGATCTTACGTAGCCCTGACT

GACTCATGGAAATCCTCCTACCTCTCAGGCCCACCTACCCTCTATTACTTCTAAGGAGTTTCTCACAGTCAACAGAATTA

GTGTCCTCCTGGCATGGATTTCAGAACTGACTTCATCAACCAACTTCACTGATGAGCTCTCGCGGCCGC
